# Mebendazole prevents distant organ metastases in part by decreasing ITGβ4 expression and cancer stemness

**DOI:** 10.1186/s13058-022-01591-3

**Published:** 2022-12-28

**Authors:** Natalie S. Joe, Inês Godet, Nubaira Milki, Noor U. I. Ain, Harsh H. Oza, Gregory J. Riggins, Daniele M. Gilkes

**Affiliations:** 1 Department of Oncology, The Sidney Kimmel Comprehensive Cancer Center, The Johns Hopkins University School of Medicine, Baltimore, MD 21231 USA; 2 Cellular and Molecular Medicine Program, The Johns Hopkins University School of Medicine, Baltimore, MD 21231 USA; 3 Department of Chemical and Biomolecular Engineering, The Johns Hopkins University, Baltimore, MD 21218 USA; 4 Johns Hopkins Institute for NanoBioTechnology, The Johns Hopkins University, Baltimore, MD 21218 USA; 5 NIH NIDDK Short-Term Research Experience Program to Unlock Potential (STEP-UP), The Johns Hopkins University School of Medicine, Baltimore, MD 21231 USA; 6 Department of Neurosurgery, The Johns Hopkins University School of Medicine, Baltimore, MD 21231 USA

**Keywords:** Mebendazole, Triple-negative breast cancer, Metastasis, Cancer prevention

## Abstract

Breast cancer is the most diagnosed cancer among women. Approximately 15–20% of all breast cancers are highly invasive triple-negative breast cancer (TNBC) and lack estrogen, progesterone, and ERBB2 receptors. TNBC is challenging to treat due to its aggressive nature with far fewer targeted therapies than other breast cancer subtypes. Current treatments for patients with TNBC consist of cytotoxic chemotherapies, surgery, radiation, and in some instances PARP inhibitors and immunotherapy. To advance current therapeutics, we repurposed mebendazole (MBZ), an orally available FDA-approved anthelmintic that has shown preclinical efficacy for cancers. MBZ has low toxicity in humans and efficacy in multiple cancer models including breast cancer, glioblastoma multiforme, medulloblastoma, colon cancer, pancreatic and thyroid cancer. MBZ was well-tolerated in a phase I clinical trial of adults recently diagnosed with glioma. We determined that the half-maximal inhibitory concentration (IC_50_) of MBZ in four breast cancer cell lines is well within the range reported for other types of cancer. MBZ reduced TNBC cell proliferation, induced apoptosis, and caused G2/M cell cycle arrest. MBZ reduced the size of primary tumors and prevented lung and liver metastases. In addition, we uncovered a novel mechanism of action for MBZ. We found that MBZ reduces integrin β4 (ITGβ4) expression and cancer stem cell properties. ITGβ4 has previously been implicated in promoting “cancer stemness,” which may contribute to the efficacy of MBZ. Collectively, our results contribute to a growing body of evidence suggesting that MBZ should be considered as a therapeutic to slow tumor progression and prevent metastasis.

**Supplementary Information:**

The online version contains supplementary material available at 10.1186/s13058-022-01591-3.

## Introduction

Breast cancer (BC) is the most common neoplasm among women and comprises more than 500,000 deaths worldwide, with ~ 2.0 million new cases diagnosed each year [1]. Breast cancer is characterized by molecular subtypes [2–5] and histological subtypes [6, 7]. Pathologists use immunohistochemical (IHC) staining to determine the presence or absence of two hormone receptors (HR), the progesterone receptor (PR) and the estrogen receptor (ER), as well as the human epidermal growth factor receptor 2 (HER2) [6, 7]. While there are targeted therapies for patients with HR+/HER2−, HR+/HER2+, HR−/HER2+ disease, triple-negative (TN) BC lacks HR and HER2, rendering ER and HER2 therapies ineffective [8]. TNBC is aggressive and often leads to bone, brain, liver, and lung metastases [9, 10], emphasizing that new therapeutics are critically needed to improve patient outcomes.

Mebendazole (MBZ) was approved by the US Food and Drug Administration (FDA) in 1971 as an anthelmintic drug to treat parasitic infections [11] but has recently been shown to have preclinical efficacy [12] in the treatment of pancreatic [13], lung [14, 15], thyroid [16], breast [17], meningioma [18], brain [19–21], melanoma [22], and colorectal [23] cancers. The first mechanism of action reported for MBZ was tubulin disruption. Tubulin is a common target of chemotherapy drugs such as paclitaxel, colchicine, and vincristine, which explains MBZ’s anti-proliferative effect in cancer cells [24]. Other reported tumor suppressive mechanisms of MBZ include inhibiting angiogenesis and inducing apoptosis through B cell lymphoma 2 (BCL-2) and caspase-3-dependent mechanisms [21, 25, 26]. MBZ treatment also induces G2/M cell cycle arrest and apoptosis leading to reduced lung metastasis in papillary and anaplastic thyroid cancer preclinical models [16]. In addition, a phase I clinical trial of adults with newly diagnosed high-grade glioma demonstrated that MBZ is safe and tolerable [27]. The promising results prompted us to determine whether MBZ could be used for the prevention or treatment of breast cancer metastasis.

Given the lack of treatment options and the propensity for TNBC to metastasize, we hypothesized that MBZ might be safe and effective for the prevention and treatment of metastasis. As reported for other cancer types, we find that MBZ promoted apoptosis and G2/M cell cycle arrest [13, 16] but reduced the expression of genes involved in angiogenesis and cell migration [12, 16, 19, 23, 26, 28, 29]. For the first time, we demonstrate that MBZ reduced lung metastasis and eliminated liver metastases in mouse models of TNBC. TNBC tissue has been reported to be enriched for cancer stem cells (CSCs) which contributes to the aggressive nature of the disease. Cancer cell stemness is known to be regulated by ITGβ4, and cancer stem cells (CSCs) have been shown to contribute to metastasis [30]. We show that treatment with MBZ led to a dramatic reduction in integrin β4 (ITGβ4) expression both in vitro and in cancer cells harvested from tumor-bearing mice following MBZ treatment. Taken together, our results show that mebendazole prevents distant organ metastases in TNBC models, in part by decreasing ITGβ4 expression and cancer stemness.

## Methods

### Cell lines and cell culture

Mycoplasma-free breast cancer cell lines, MDA-MB-231 (ATCC^®^ HTB-26™) and 4T1 (ATCC^®^ CRL-2539™) cells, were obtained from American Type Culture Collection (ATCC) and maintained in DMEM (MDA-MB-231; Sigma-Aldrich) or RPMI-1640 (4T1; Sigma-Aldrich) with 10% fetal bovine serum (FBS; Corning) and 1% penicillin/streptomycin (P/S) (Invitrogen). SUM159 and 4T1-luciferase-tagged (4T1-Luc) cells were kindly provided by the Sukumar lab and were cultured in Ham’s F12 medium supplemented with 5% FBS, 1% P/S, and 5% insulin/hydrocortisone (SUM159) or in DMEM with 10% FBS and 1% P/S (4T1-Luc). Cells were maintained in a humidified environment at 37 °C and 5% CO_2_. MMTV-PyMT cells were derived from a tumor excised from a female triple-transgenic mouse as previously described [31] and maintained in 50% DMEM, 50% DMEM/F12, 10% FBS, 5% insulin, and 1% P/S. Fluorescent MDA-MB-231 cells were developed and maintained as previously described. [32]

### Proliferation assays

Half-maximal inhibitory concentration (IC_50_) values were obtained for MMTV-PyMT, MDA-MB-231, 4T1, and SUM159 cells by seeding 1000 cells/well in a 96-well plate for 24 h (h) and then treated with 0.01–500 µM of mebendazole (MBZ) or less than 1% dimethyl sulfoxide (DMSO) as vehicle control. After 48 h of treatment, PrestoBlue (Thermo Fisher) was added to achieve a 10% (v/v) concentration in each well, incubated for 4 h, and fluorescence was measured using a Cytation5 (BioTek Instruments). The IC_50_ was calculated using a nonlinear fit log vs. response model. To calculate % cell survival, cells were fixed with 70% ethanol, stained with DAPI, and imaged using a Cytation 5 (BioTek Instruments) equipped with an Olympus–UPLFLN 4XPh phase objective and DAPI filter. A 4 × 3 montage was used to capture the entire area of each well, and NIS Elements software (Nikon Instruments Inc.) was used to threshold the DAPI positive area of the image which is presented as % survival.

### Colony formation assays

MDA-MB-231, 4T1, and SUM159 cells were plated in a 24-well plate (250 cells/well) and exposed to 0.01–1 µM of MBZ or < 1% DMSO as vehicle control for 10–14 days (refreshed every 3 days) or pre-treated with MBZ for 48 h and then seeded in plates without further drug treatment. The cells were washed with 1 × phosphate-buffered-saline (PBS), fixed with 4% paraformaldehyde (PFA) in PBS for 15 min (min), and washed again with PBS. Crystal violet solution (1% (w/v) crystal violet diluted in water containing 20% methanol) was added to each well and incubated for 10 min at room temperature. Wells were washed with distilled water and dried. Well plates were imaged using a Cytation 5 (BioTek Instruments) equipped with an Olympus–UPLFLN 4XPh phase objective in color bright field. A 4 × 3 montage of the acquired images was used to capture an image of the entire well. Colonies were quantified by manually counting individual colonies in each well.

### Cell cycle analysis

MDA-MB-231 cells were treated with 0.25 µM, 0.5 µM, 1 µM, and 5 µM of MBZ or DMSO (vehicle control) for 48 h, washed with PBS, fixed in 70% ethanol, and pelleted and stained with 100 µg/mL RNase and 50 µg/mL of propidium iodide in the dark at 4 °C overnight. The distribution of cells in G1, S, and G2/M phases was determined by flow cytometry using a CytoFLEX flow cytometer (Beckman Coulter), and data analysis was performed using FlowJo software V10.

### Western blotting

Cell homogenates were prepared from MDA-MB-231 and SUM159 cell lines or primary tumors formed by MDA-MB-231 cells lysed in IGEPAL CA-630 buffer (150 mM sodium chloride (NaCl), 1% IGEPAL CA-630, 50 mM Tris–HCL, pH 8.0, protease and phosphatase inhibitors) for 10 m on ice. The lysate was centrifuged for 10 min at 13,000 g at 4 °C, and the supernatant was collected. Proteins were fractionated by a 10% sodium dodecyl sulfate–polyacrylamide gel electrophoresis (SDS-PAGE) and then transferred to a nitrocellulose membrane for 30 min using a Trans-blot Turbo (Bio-Rad). The membranes were blocked in 5% milk (% w/v) in 1 × Tris-buffered saline and 0.1% Tween-20 (TBS-T) and incubated overnight in primary antibodies at a dilution of 1:1000 at 4 °C. The membranes were washed three times in TBS-T and incubated in HRP-conjugated secondary antibodies (Cell Signaling Technology) followed by three additional washes in TBS-T. The chemiluminescence signal was detected using an AZURE C300 (Azure™ Biosystems) after incubating the membrane with ECL (Perkin Elmer). A list of antibodies can be found in Additional file 1: Table S1.

### Transwell migration assays

Transwell invasion assays were performed using Costar Transwell cell culture inserts (Corning) with an 8-μm pore size PET membrane in a 24-well plate. The lower chambers of the transwell plates were filled with 500 μL of cell-appropriate culture media. MDA-MB-231 or SUM159 cells were pre-treated with 0.5 or 1 μM of MBZ for 48 h. The cells (20,000/well) were resuspended in 150 μL of medium containing only 1% FBS and placed into the upper well. After 24 h, cells in the upper chamber were removed with a cotton swab, and those that migrated through the pores on the lower surface were fixed in 100% ethanol (EtOH) and stained in 0.2% crystal violet dye. Five random fields of each pore were imaged using a Cytation 5 (BioTek Instruments) equipped with an Olympus–UPLFLN 10XPh phase objective. For MDA-MB-231 cells, the percent area and total area were quantified using ImageJ software using a custom macro with the following steps: Each image was converted to 16-bit, the scale was converted from pixel to μm, the background was subtracted using a rolling ball radius of 10 pixels and light background, and a threshold was set to highlight crystal violet positive areas of the wells. The macro was altered to include the color threshold feature, the “hue” slider was used to highlight the cell area, and images were processed at 8-bit to quantify the % area for SUM159 cells.

### Wound-healing assays

MDA-MB-231 and SUM159 cells were cultured in a 12-well plate (100,000 cells/well) and allowed to reach near confluence within 48 h. Linear scratches of equal width were introduced using a 200 µL pipette tip. Fresh media containing 0.25 µM, 0.35 µM, 0.5 µM MBZ, or DMSO (vehicle control) was added. Five images of the wounded area were taken at 12 and 24 h using a Cytation 5 (BioTek Instruments) equipped with an Olympus–UPLFLN 4XPh phase objective. A montage of the entire well was constructed by stitching individual images. The average healing speed and area covered were quantified using ImageJ software. The following macro was created: Images were converted to an 8-bit format, scale was set with the appropriate pixel to μm conversion, a Gaussian blur of radium 5 pixels was applied, and a threshold was applied using the measure tool to highlight only the gap created by the wound. The macro code calculates the wound area covered in μm^2^; this value was then divided by the total hours of the experiment (12 h or 24 h).

### DNA extraction from mouse tissues

Mouse liver or lung tissues were placed in Genomic DNA lysis buffer (1 M Tris, pH 8.0, 5 M NaCl, 0.5 M EDTA, 10% Tween-20, 10% NP-40, and 40 μg of proteinase K) at 55 °C for 3 h with intermittent vortex. Proteinase K was inactivated with a 2 min incubation at 95 °C. One volume of phenol/chloroform/isoamyl alcohol (25:24:1) (Sigma) was added to each sample, followed by 20 s vortex. Samples were centrifuged for 10 min at 13,000 × g, and the upper aqueous phase was collected. Glycogen (20 μg), 0.1 volume of 2.5 M NaCl, and 2 × volume of 100% EtOH were added, and samples were incubated for 20 min at − 20 °C. The samples were spun at 13,000 × g for 15 min to pellet the DNA followed by one wash with 70% EtOH. The sample was spun at 13,000 × g for 5 min, the remaining liquid was decanted, and the DNA pellets were air-dried. DNA pellets were resuspended in DNase/RNase-free water. To assess the amount of human DNA content in the mouse lung and liver, the cycle threshold value of human hexokinase 2 (HK2) measured by RT-PCR was normalized to the DNA concentration of the sample (ranged from 180 to 220 ng/µL). The result for each sample was than normalized to the average value of the control group.

### Reverse transcription and quantitative polymerase chain reaction (qPCR)

**Fig. 1:**
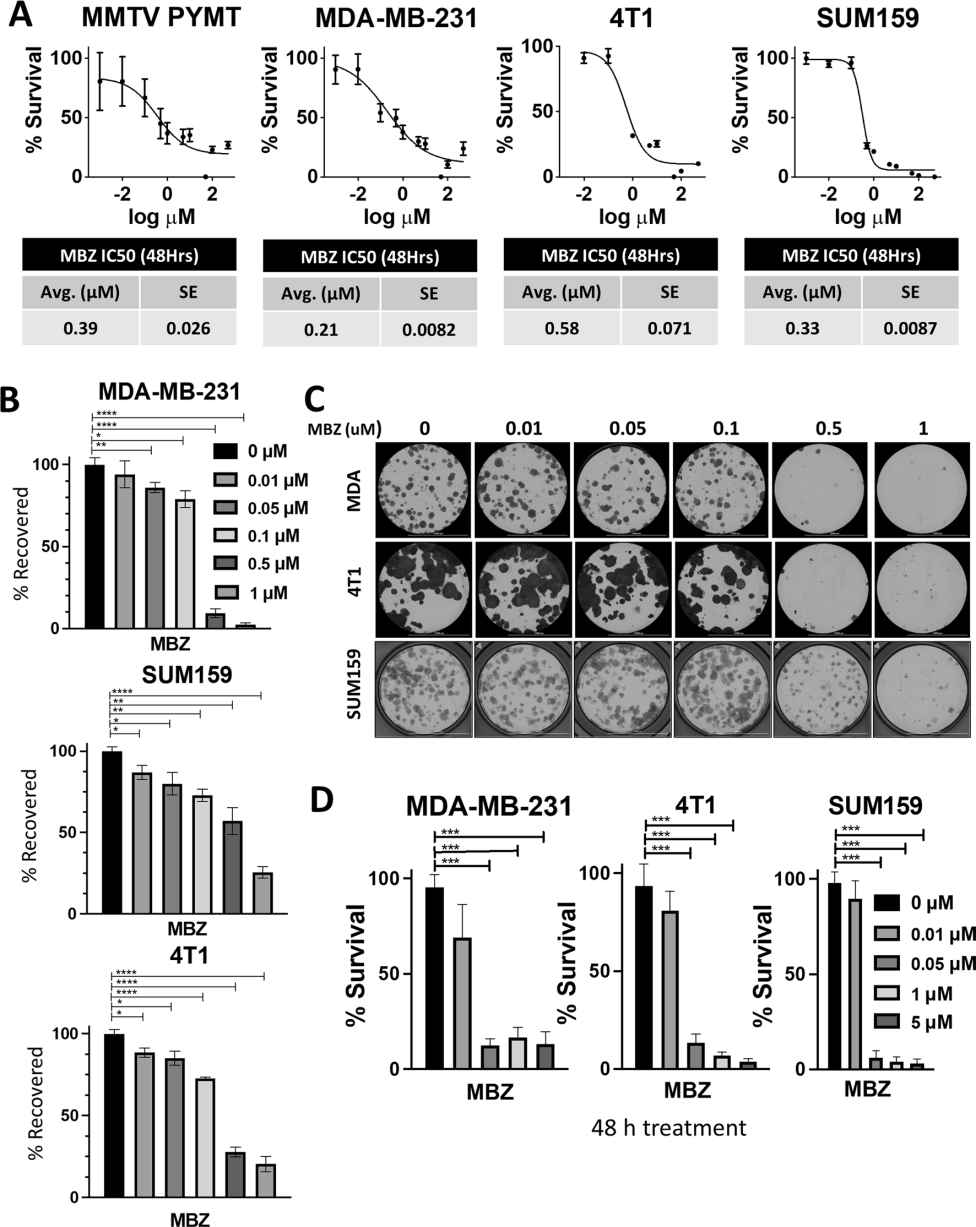
Mebendazole (MBZ) inhibits the proliferation of triple-negative breast cancer (TNBC) cells. **A** The half-maximal inhibitory concentration (IC_50_) of MBZ in MMTV-PYMT, MDA-MB-231, 4T1, and SUM159 cells is plotted with standard error (SE) represented by bars. **B** MDA-MB-231, 4T1, and SUM159 cells were pre-treated for 48 h with MBZ at varying concentrations and allowed to recover for 10–14 days in growth media. Colonies were stained with crystal violet and quantified to determine the percentage area of the well covered by crystal violet (% recovered). Mean ± SEM; *N* = 3 independent experiments. *P* values for one-way ANOVA test *< 0.05, **< 0.01, ***< 0.001,****< 0.0001. **C** Representative images of colony formation described in (**B**). **D** MDA-MB-231, 4T1, and SUM159 cells were seeded at 1000 cells/well in a 96-well plate for 24 h, treated with various concentrations of MBZ for 48 h, and stained with DAPI. The % DAPI area (see Additional file 2: Fig. S1C) was quantified and presented as % survival. Mean ± SEM; *N* = 3 independent experiments with *n* = 4 technical replicates. *P* values for one-way ANOVA test *< 0.05, **< 0.01,***< 0.001

**Fig. 2:**
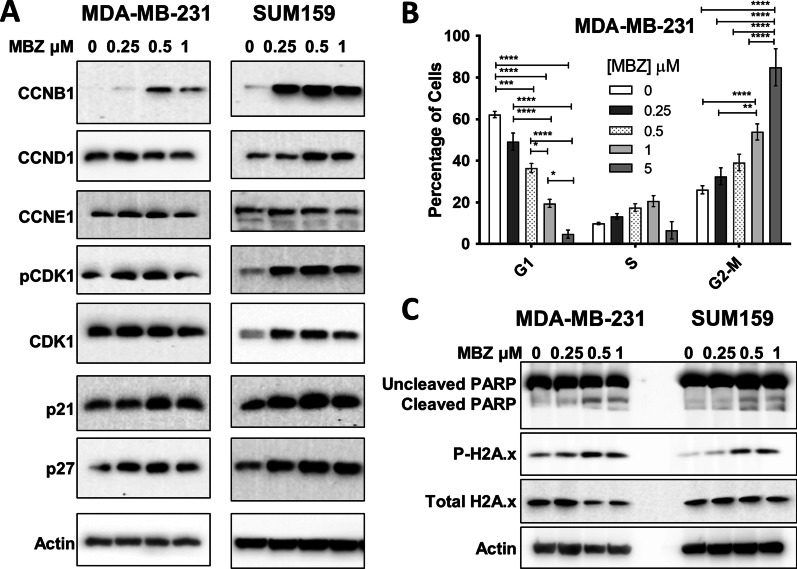
Mebendazole induces G2/M cell cycle arrest and apoptosis. **A** Expression of proteins involved in G2/M cell cycle arrest was analyzed by Western blot using lysate extracted from MDA-MB-231 (left) and SUM159 (right) cell lines treated with increasing doses of MBZ as indicated. **B** The percentage of MDA-MB-231 cells in each phase of cell cycle was determined after treating the cells for 48 h with MBZ at the indicated doses followed by propidium iodide staining and flow cytometry analysis. Mean ± SEM; *N* = 3 independent experiments. *P* values for two-way ANOVA test *< 0.05, **< 0.01,***< 0.001,****< 0.0001. **C** Expression of proteins involved in MBZ-induced apoptosis was analyzed by Western blot using lysate extracted from MDA-MB-231 and SUM159 cells treated with increasing doses of MBZ for 72 h

**Fig. 3:**
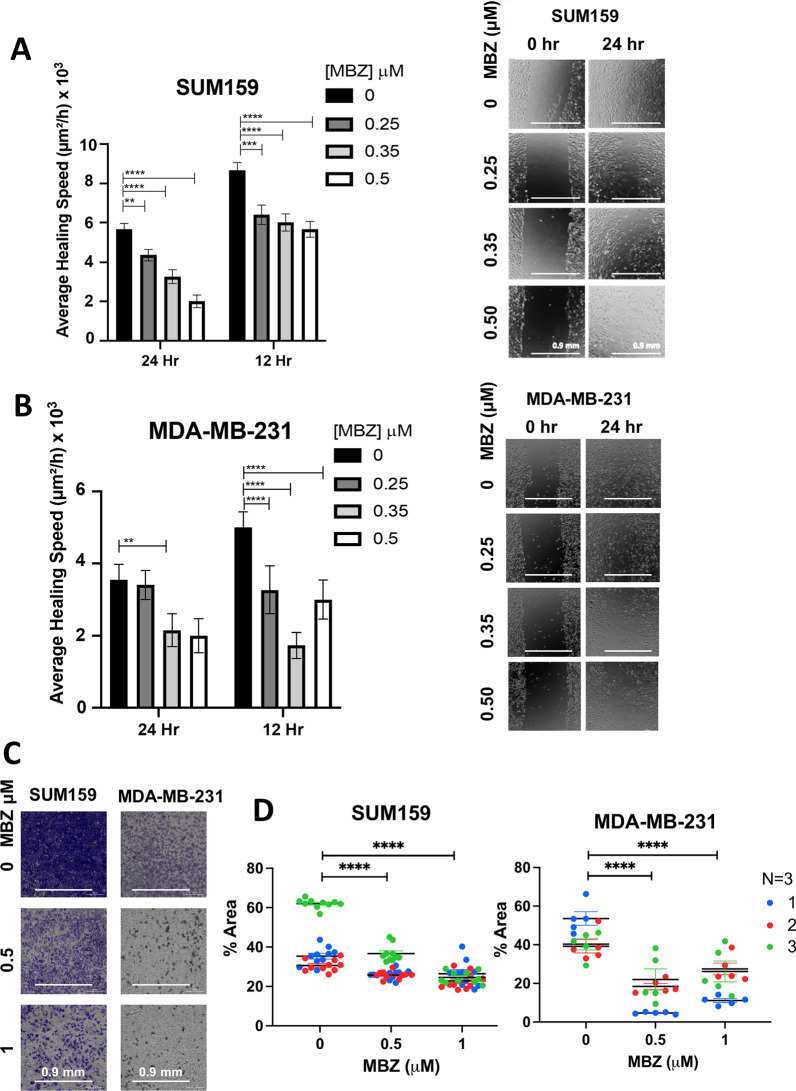
Mebendazole reduces the migration of TNBC cells. **A**, **B** Quantification of relative wound closure of SUM159 **A** and MDA-MB-231 **B** cells treated for 12 h and 24 h with indicated concentrations of MBZ or DMSO as a vehicle control. Corresponding representative photomicrographs are shown at the right **A** mean ± SEM; *N* = 3 independent experiments. *P* values for two-way ANOVA test comparing vehicle control to each individual treatment dose *< 0.05, **< 0.01, ***< 0.001, ****< 0.0001. **B** Scale bar = .9 mm. **C**, **D** MDA-MB-231 and SUM159 cells were pre-treated for 48 h with 0.5 μM or 1 μM of MBZ or DMSO and plated for transwell assays. The number of cells that migrated through the filter was determined by crystal violet staining followed by bright-field imaging (**C**) and quantified by the percent area of crystal violet staining (**D**). Mean ± SEM; *N* = 3 independent experiments. *P* values for two-way ANOVA test comparing vehicle control to each individual treatment dose **< 0.01,***< 0.001,****< 0.0001

**Fig. 4:**
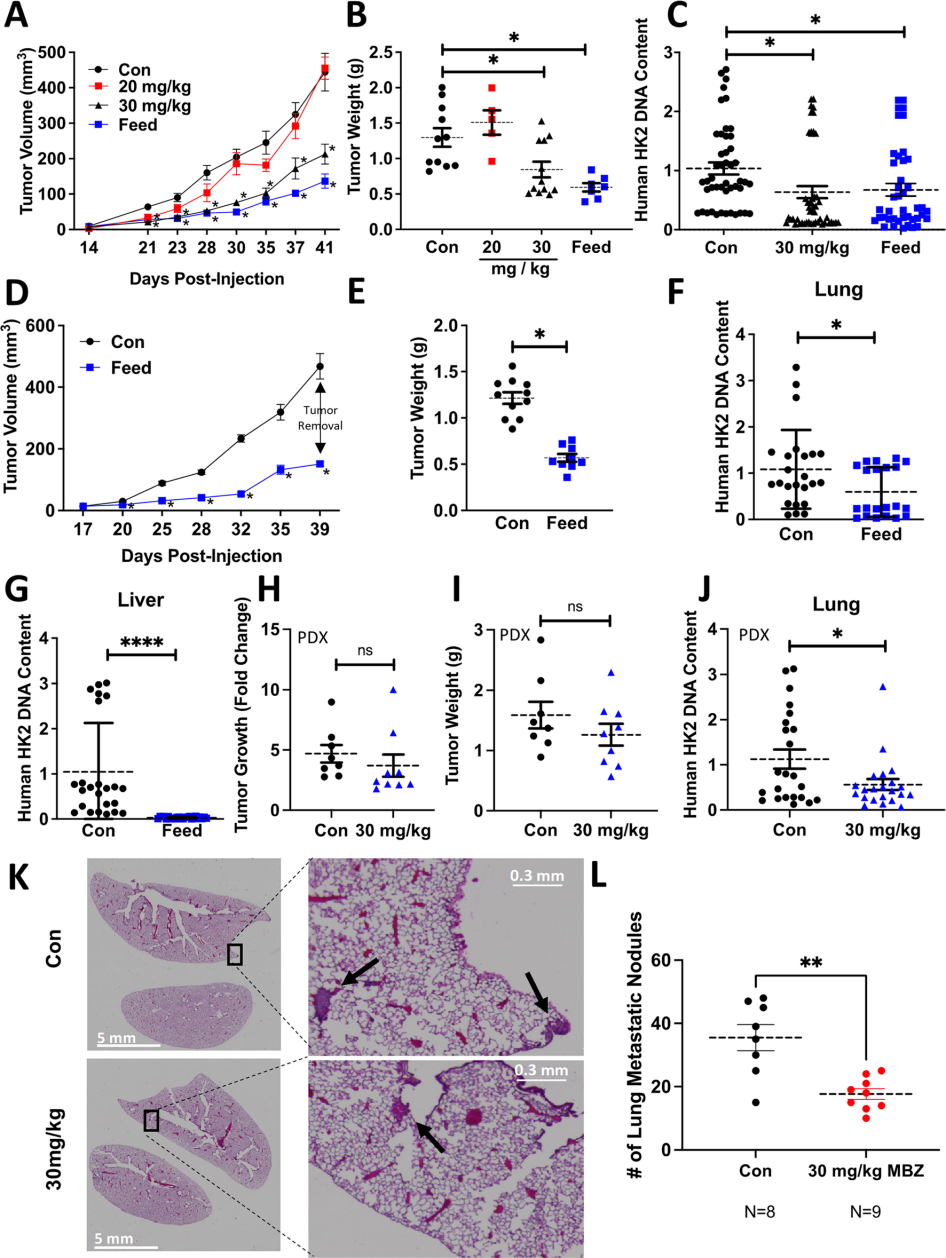
Mebendazole reduces tumor growth and lung and liver metastases. **A** Fluorescently labeled MDA-MB-231 cells were implanted into the mammary fat pad of NSG mice. Mice were treated beginning on day 5 with sesame oil as a vehicle gavage and a high-fat KetoCal diet (Con; *N* = 12), 215 ppm MBZ incorporated into a high-fat KetoCal diet (Feed; *N* = 7), 20 mg/kg MBZ in a sesame oil gavage (20 mg/kg; *N* = 6), 30 mg/kg MBZ in a sesame oil gavage (30 mg/kg; *N* = 12). Mice were exposed to the feed seven days a week, and treatments administered via oral gavage were given four times per week. Tumor volumes are plotted over time. *P* values for two-tailed *t* test *< 0.05. **B** Tumor weights for each treatment group were taken at the endpoint of the experiment (41 days after implantation). *P* values for two-tailed *t* test *< 0.05. **C** Metastatic burden was determined by measuring human genomic HK2 DNA content in the lung of each mouse normalized to their final tumor weight. The final values were normalized by the average of the Con group (mean ± SEM *N* = 6–12 mice with *n* = 3 technical replicates). *P* values for two-tailed *t* test ****< 0.0001. **D** Tumor growth curves displaying tumor volume over time prior to tumor removal at Day 39 (mean ± SEM) for NSG mice that began treatment on day 5 with either a high-fat KetoCal diet (Con; *N* = 11) or 215 ppm of MBZ incorporated into a high-fat KetoCal diet (feed; *N* = 10). *P* values for two-tailed *t* test *< 0.05. **E** Final tumor weights at the time of tumor removal, 39 days after implantation (mean ± SEM *N* = 10–11 mice). *P* values for two-tailed *t* test *< 0.05. **F**, **G** Metastatic burden was determined by measuring human genomic HK2 DNA content in mouse lung **F** or liver **G** two-week post-tumor removal surgery. Values were normalized to individual tumor weight and then to the average value of the control group (mean ± SEM, *N* = 10–11 mice with *n* = 3 technical replicates). *P* values for paired two-tailed *t* test *< 0.05, ****< 0.0001. **H** Quantification of tumor growth rate (fold change of final tumor volume versus tumor volume at the start of treatment) over two weeks for NSG mice bearing primary HCI-001 PDX tumors. Mice were treated 4 times per week with 30 mg/kg MBZ in sesame oil (*N* = 9) or sesame oil alone (*N* = 8) by oral gavage. Two-way paired Student’s *t* test. **I** Final tumor weight of HCI-001 PDX primary tumors described in (**H**). **J** Metastatic burden was determined as described in (F-G). (Mean ± SEM *N* = 8–9 mice with *n* = 3 technical replicates). *P* values for two-tailed Student’s *t* test * < 0.05. **K** Representative lung sections stained with hematoxylin and eosin (H&E) from NSG mice described in 4H. **L** Quantification of lung metastatic nodules pictured in (K). Mean ± SEM; control (*N* = 8 mice) and 30 mg/kg MBZ (*N* = 9 mice). *P* values for paired two-way *t* test **< 0.01

**Fig. 5:**
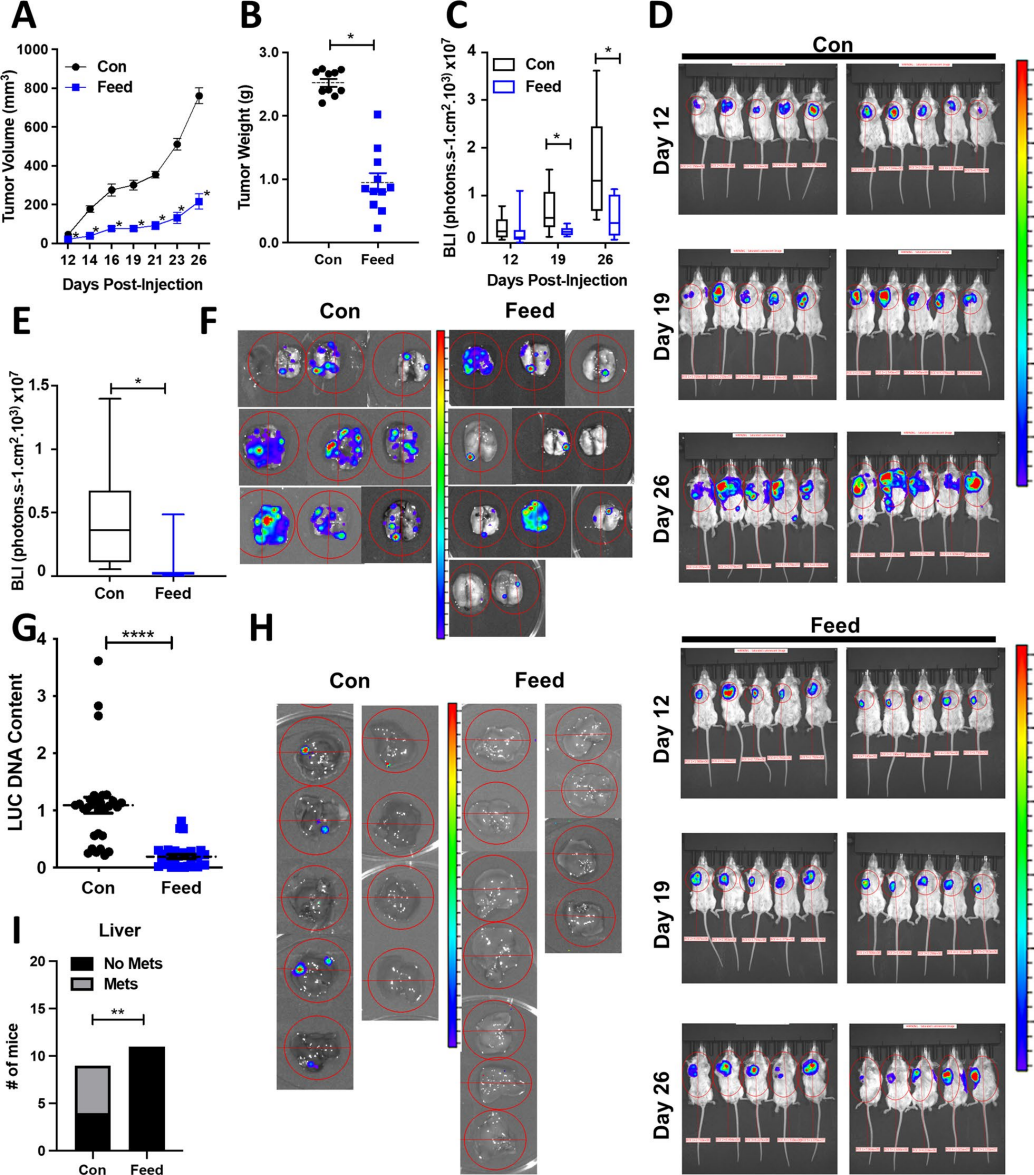
Mebendazole reduces tumor growth and lung and liver metastases in an immune-competent animal model of metastasis. **A** Luciferase-labeled 4T1-cells were implanted into the mammary fat pad of Balb/c mice. Tumor growth curves displaying tumor volume over time are plotted for each treatment group (mean ± SEM, *N* = 10–11 mice). Treatments included a high-fat KetoCal diet (Con; *N* = 10) or 250 ppm of MBZ incorporated into a high-fat KetoCal diet (Feed; *N* = 11). *P* values for two-tailed *t* test *< 0.05. **B** Final tumor weights are plotted for mice in each treatment group. *P* values for two-tailed *t* test * < 0.05. **C**, **D** The bioluminescence intensity (BLI) of each tumor was imaged and quantified on day 12, 19, and 26 in live mice. *P* values for two-tailed *t* test *< 0.05. **E** The BLI of each mouse lung was quantified on the final day of the experiment after resecting (day 26). *P* values for two-tailed *t* test * < 0.05. **F** BLI images of lung metastasis prior to DNA extraction. **G** Lung metastatic burden was quantified by measuring the DNA content of the luciferase (LUC) gene in each mouse lung divided by individual tumor weights and then normalized to the average value of the control group (mean ± SEM, *N* = 10–11 mice with *n* = 3 technical replicates). *P* values for two-tailed *t* test ****< 0.0001. **H** BLI images of liver metastasis prior to DNA extraction. **I** The total number of mice with liver metastasis based on a bioluminescence threshold of 4000 photons.s^−1^ cm^2^ is displayed. Chi-square test **< 0.01

Total RNA was extracted using TRIzol (Invitrogen) followed by cDNA synthesis using Promega’s GoScript™ Reverse Transcriptase. qPCR analysis was conducted on CFX96 Real-Time PCR detection system (Bio-Rad) using SYBR Green qPCR master mix (Bio-Rad). The expression of each target mRNA relative to 18S rRNA control was calculated based on the cycle threshold (Ct) as 2 − Δ(ΔCt), in which ΔCt = Ct (target mRNA) − Ct (18S rRNA), and Δ(ΔCt) = ΔCt (treatment) − ΔCt (control). Primer sequences are listed in Additional file 1: Table S2. (Figs. 1, 2, 3, 4, and 5).

### RNA sample preparation and sequencing

**Fig. 6:**
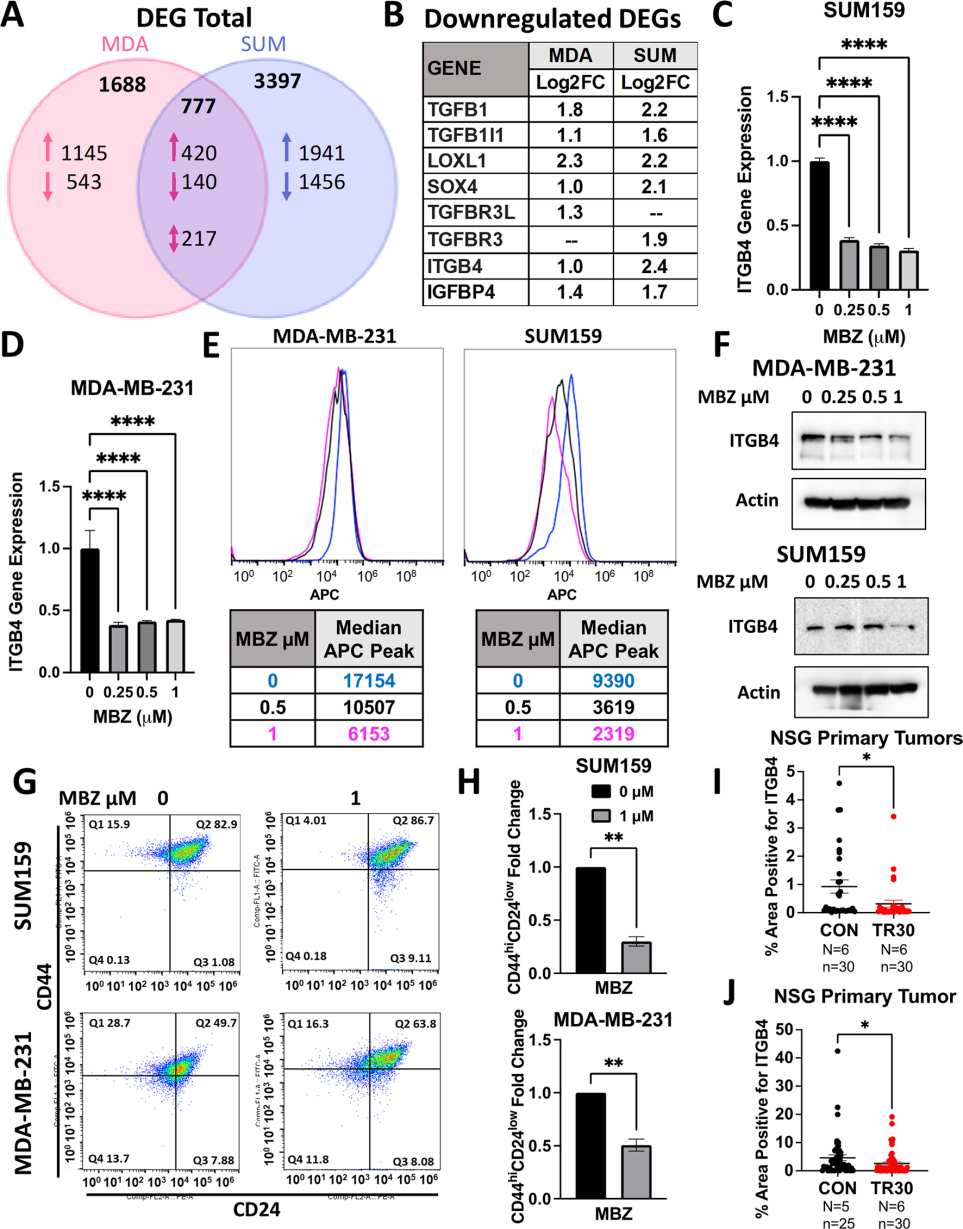
Mebendazole treatment reduces ITGβ4 expression. **A** Venn diagram displaying the number of genes with differential expression following MBZ treatment (− 2 ≥ FC ≥ 2) that overlap or are exclusive to MDA-MB-231 (pink circle) and SUM159 (blue circle) cells. Additional file 3 includes the gene lists used to generate the VENN Diagram. **B** A table representing DEGs that are downregulated in both MDA-MB-231 and SUM159 cells and that have been implicated in cancer metastasis is presented with individual log2 fold change from the RNA sequencing analysis **C**, **D** Quantification of ITGβ4 relative gene expression from RT-qPCRs performed with SUM159 **C** and MDA-MB-231 **D** cells treated for 72 h with indicated doses of MBZ or DMSO. Mean ± SEM; *N* = 3 independent experiments with *n* = 3 technical replicates. *P* values for one-way ANOVA test ****< 0.0001. **E** Representative histograms for ITGβ4 expression in MDA-MB-231 and SUM159 cells as measured by flow cytometry after 72 h of treatment with 0.5 μM and 1 μM MBZ or DMSO. The APC peak median values are listed. **F** Expression of ITGβ4 protein analyzed by Western blot using lysate extracted from MDA-MB-231 and SUM159 cell lines treated with increasing doses of MBZ as indicated or DMSO. **G** Representative FACS analysis of the ITGβ4 + MDA-MB-231 and SUM159 cells that were co-stained with CD44 and CD24 antibodies after being treated with 1 μM of MBZ or DMSO for 72 h. **H** The fold change of CD44^hi^CD24^low^cells within the ITGβ4+ gated population of SUM159 and MDA-MB-231 cells described in (G). Mean ± SEM; *N* = 3 independent experiment. *P* values for paired two-way *t* test **< 0.01 **I**, **J** Quantification of immunofluorescent staining for ITGβ4 protein in tissue sections from mouse tumors (Fig. 4) treated with either 30 mg/kg MBZ in sesame oil suspension (*N* = 6) via gavage or vehicle control (sesame oil alone; *N* = 6) with the primary tumor onboard (**I**) and tumor removal (**J**). Mean ± SEM; *N* = 6 or 5 independent mice per group with *n* = 5 technical replicates for percent positive area quantification. *P* values for two-way *t* test * < 0.05

MDA-MB-231 and SUM159 cells were collected from culture, and subsequently, total RNA was extracted. Following RNA purification, samples were confirmed to have a RIN value > 9.0 using an Agilent Bioanalyzer. Total RNA was further qualified upon receipt to Novogene (Sacramento, CA). The samples were assessed using the RNA Nano 6000 Assay Kit of the Bioanalyzer 2100 system (Agilent Technologies, CA, USA). RNA purity was checked using the NanoPhotometer spectrophotometer (IMPLEN, CA, USA). One μg of RNA was used as input for mRNA library preparation. mRNA was purified from total RNA using poly-T oligo-attached magnetic beads followed by double-stranded cDNA synthesis. The cDNA fragments were adenylated, and sequencing adaptors were added. Each 150–200 bp length fragment was purified, and PCR was performed per Novogene established protocols. Sequencing was performed on a NovaSeq 6000 system with 150-bp paired-end run by Novogene (Sacramento, CA). The reads were mapped to Homo Sapiens (GRCh38/hg38) using STAR (v2.5) with the parameter mismatch set to 2. Quantification was done using HTSeq (v0.6.1) software with the parameter -m union. Differentially expressed genes were identified using EdgeR (v3.16.5) with padj < 0.005 and [log2(FoldChange)] > 1. FASTQ files and read counts have been uploaded to GEO with the accession number: GSE190845. For the gene ontology enrichment analysis, the *p* value cutoff was set at *p* < 0.05. In order for a GO pathway to be included in Additional file 2: Fig. S5, the pathway had to be enriched (*p* < 0.05) in both the SUM159 and MDA-MB-231 cell lines. Differentially expressed genes (DEGs) consisted of genes that increased or decreased with a fold change of 1.5 or higher. Genes meeting this criteria were included in the VENN diagram in Fig. 6. Additional file 3 (SuppDataRNAseq.xls) includes all GO enrichment scores and differential expression values for each cell line.

### Immunofluorescence staining

Immunofluorescence staining to detect ITGβ4 was performed on 10-μm-thick tissue cryo-sections after rehydrating them by immersing them into 1 × phosphate-buffered saline and 0.1% Tween-20 (PBS-T). All slides were then blocked with 2% BSA for 60 min and incubated overnight at 4 °C with a primary antibody against ITGβ4 (50–1049-80, Invitrogen) (dilution at 1:200). The next day, slides were washed 3× with PBS-T and then incubated with DAPI (1 ug/mL) for 15 m at room temperature. Sudan Black (0.1% w/v) was applied to the slides for 25 min to quench autofluorescence followed by washing in PBS-T. ITGβ4 and DAPI were visualized in Cy5 and DAPI channels, respectively, using Cytation 5 (BioTek Instruments) equipped with an Olympus–UPLFLN 10XPh phase objective.

### Mammosphere formation assay

Fluorescently tagged MDA-MB-231 cells in culture or freshly resected from tumors that were processed (enzymatic digestion in 2 mg/mL collagenase for 1 h at 37 °C with orbital shaking at 160 rpm) were passed through a cell strainer (70 μm) and plated at 2,000 cells/well in 12-well plates previously coated with polyHema (12 g/L in 95% EtOH) and air-dried for 48 h, with 2 mL of mammosphere formation media per well (MammoCult basal medium (human) containing 4 μg/mL heparin, and 0.48 μg/mL hydrocortisone) (STEMCELL Technologies; Vancouver, BC, Canada). Fluorescent images were captured using a Cytation 5 (BioTek Instruments) equipped with an Olympus–UPLFLN 4XPh phase objective. A montage of the entire well was constructed by stitching individual images. The total number and average size of each mammosphere were then calculated using Gen5 software (BioTek instruments) automated counting algorithm.

### Fluorescence-activated cell sorting (FACS)

MDA-MB-231 and SUM159 cells were treated with fresh media containing 0.05 µM, 0.125 µM, 0.5 µM, 1 µM, or 5 µM MBZ or DMSO (vehicle control) for 72 h. Cells were then trypsinized, resuspended in culture media, washed with 1xPBS, and collected in FACS buffer (1xPBS, 1%BSA, 0.5 mM EDTA, and 25 μg/ml DNase). Live cells were stained with APC-conjugated antibody against ITGβ4 (50-1049-80, Invitrogen) diluted at 1:300 for 30 min on ice. Sytox™ Blue (Invitrogen, dilution 1:300) was added immediately before analyzing each sample using flow cytometry. APC (ITGβ4) was detected in the FL-4 channel, and FITC (Sytox™ Blue) was detected in FL-1 channel. Or cells were stained with conjugated antibodies against ITGβ4, CD44, and CD24 diluted at 1:300 for 30 min on ice. APC (ITGβ4) was detected in the FL-4, FITC (CD44) was detected in FL-1 channel, and PE (CD24) was detected in FL-2 channel. Single stained cells were used for compensation controls. Data were analyzed using FlowJo V10 software (Tree Star, Inc.). For primary tumors that were sorted, tumors were subjected to enzymatic digestion prior to staining and ultimately resuspended in sorting buffer (1xPBS, 1%BSA, 0.5 mM EDTA, and 25 μg/ml DNase). Samples were sorted on an SH800 cytometer (Sony) into ITGβ4+ or ITGβ4− expressing populations directly into media. APC (ITGβ4) was detected in the APC channel, and Alexa Fluor 405 (Sytox™ Blue) was detected in Pacific Blue channel. Single stained cells were utilized as compensation controls. A list of antibodies can be found in Additional file 1: Table S1.

### Breast cancer patient-derived xenograft (PDX) maintenance

The HCI-001 PDX was kindly provided by the Zahnow Lab and developed by the Welm lab (https://uofuhealth.utah.edu/huntsman/labs/welm-labs/research.php). The individual demographical data can be found in the Baylor College of Medicine PDX Portal (https://pdxportal.research.bcm.edu/). The tumor fragments from the HCI-001 PDX were maintained in NOD-SCID Gamma (NSG) mice. When tumors reached the maximum diameter approved by Johns Hopkins University Animal Care and Use Committee (ACUC), they were collected and re-implanted into new mice as ~ 1 mm size fragments or cryopreserved. The initial tumor was termed “p.x+1,” and passages were tracked. For experiments, primary tumors were manually dissociated and incubated for 1 h at 37 °C in 2 mg/mL collagenase on an orbital shaker and then digested with DNase (0.4 U/ml) (Sigma-Aldrich) for 5 min at room temperature. Next, the tumor-dissociated cell suspension containing organoids was centrifuged and resuspended in fresh media followed by differential centrifugation (×4) at 520 g for 2 s. Finally, the cell suspension was strained through a 100 μm Nylon filter, counted, and then, 5000 organoids were re-implanted into each recipient NSG mouse. This process has been described in-depth previously [33].

### In vivo* orthotopic breast cancer models*

All animal research complied with relevant ethical regulations within protocols approved by the Johns Hopkins University ACUC. Female 5- to 7-week-old NOD-SCID Gamma (NSG) mice or BALB/c mice were anesthetized by the intraperitoneal injection (i.p.) of 100 mg/kg ketamine and 16 mg/kg xylazine. MDA-MB-231 cells (2 × 10^6^; NSG), 4T1-luciferase-tagged cells (500; BALB/c), or HCI-001 tumor organoids (5000; NSG) were injected into the mammary fat pad closest to the second nipple. Tumor removal surgery was performed 5.5 weeks (MDA-MB-231 model only) post-tumor implantation as described previously [34]. At the end of the experiment, tumors, livers, and lungs were excised. A portion of each organ was first formalin-fixed (Sigma-Aldrich) overnight and then saturated in 30% sucrose (Sigma-Aldrich) at 4 °C overnight. Each organ was then placed into a cryomold and covered completely with OCT media (Fisher Scientific). After flash freezing the OCT embedded organ in liquid nitrogen, they were sectioned onto Superfrost Plus Microscope Slides (Fisher Scientific) using a cryotome CM11000 (Leica). A second portion of the same tumor, liver, and lung was flash-frozen and processed for DNA or RNA extraction. Primer sequences are listed in Additional file 1: Table S2. For mice injected with the HCI-001 PDX, rather than embedding in OCT, the lungs from NSG mice were inflated with agarose, formalin-fixed, embedded in paraffin and H&E stained.

### Bioluminescence assay to measure metastatic burden

Intratumoral bioluminescence was measured weekly throughout the study by injecting the BALB/c mice bearing 4T1-Luciferase expressing tumors with 2 µg/mouse (150 µL) of D-luciferin potassium salt (Gold Bio) diluted in 1 × PBS. Mice were then briefly anesthetized with Fluorido (Isoflurane, USP) and imaged using a Xenogen imaging system (IVIS 200, equipped with Living Image Software) within 10 min of luciferin injection, as described previously [20]. All tumor bioluminescence values were expressed as radiance (photons.s-1.cm^2^.10^3^). At the end of the study, lungs and livers were excised and immediately imaged to detect bioluminescence.

### Limiting dilution assays (LDA)

**Fig. 7:**
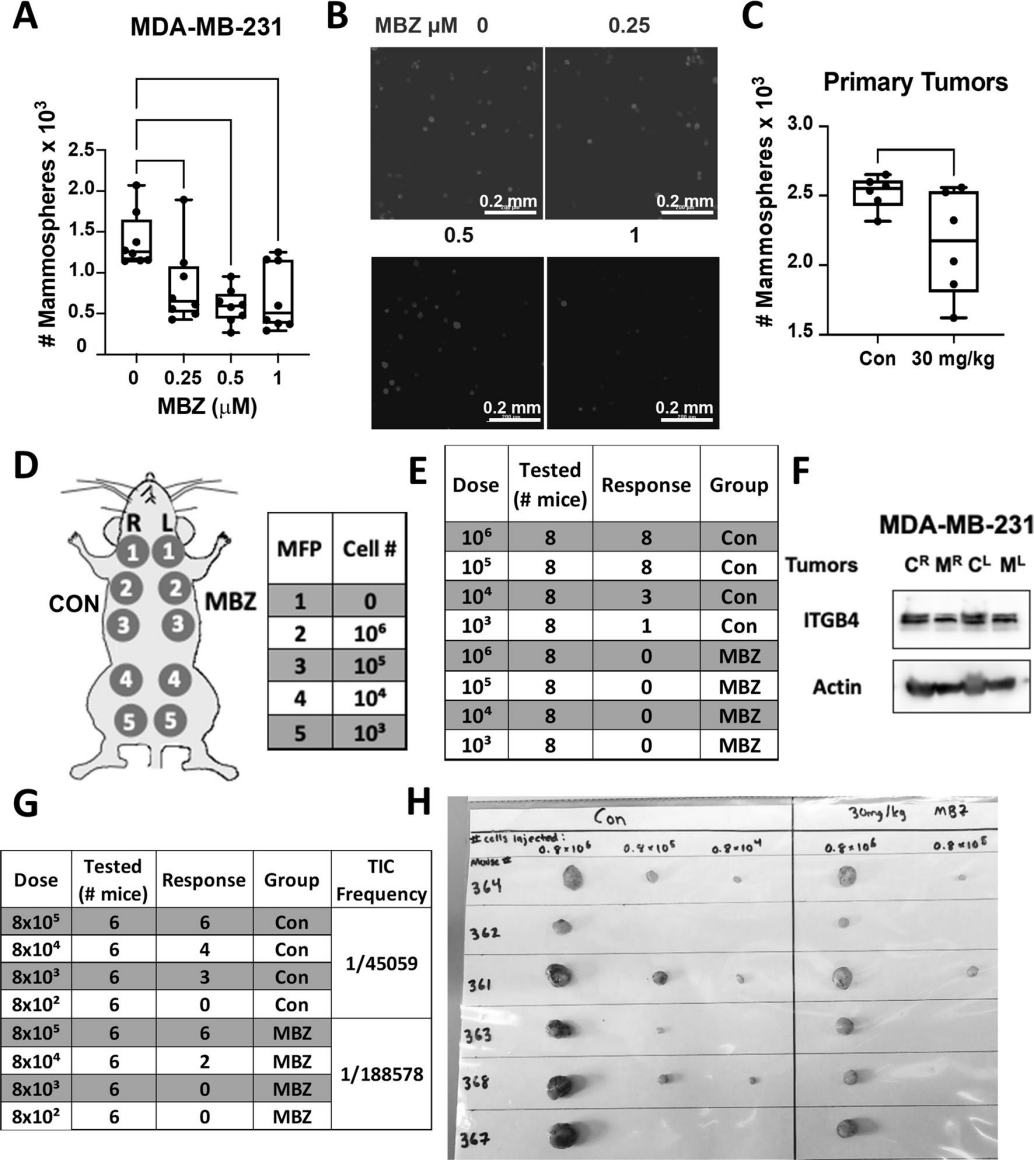
Mebendazole treatment reduces the cancer stem cell phenotype of TNBC cells. **A** Quantification of the total number of mammospheres that were formed by MDA-MB-231 cells after 72 h pre-treatment with the indicated doses of MBZ or DMSO. Mean ± SEM; *N* = 3 independent experiments with *n* = 3 technical replicates. *P* values for one-way ANOVA test *< 0.05, **< 0.01. **B** Representative images of fluorescently tagged MDA-MB-231 mammospheres quantified in (**A**). **C** MDA-MB-231 cells were harvested from tumors that were developed in mice that were treated four times per week with 30 mg/kg MBZ in a sesame oil suspension or sesame oil as vehicle control for 30 days, beginning on day 5 post-injection. The total number of mammospheres formed by these cells after 7 days of culture in mammosphere formation media was quantified. Mean ± SEM; *n* = 6 technical replicates. *P* values for two-way *t* test *< 0.05. **D** Schematic of the experimental setup used for the in vivo limiting dilution assay depicting the location of each injection site with reference to the mouse mammary fat pad. The number of cells injected per site (cell #) is listed. **E** Tumor incidence (response) at week 6 following the limited dilution and injection of MDA-MB-231 cells (dose) treated as described in 7A. **F** Mice bearing two MDA-MB-231 tumors (right^R^ and left^L^) were treated with 30 mg/kg of MBZ in a sesame oil suspension (M) or with sesame oil alone (**C**). The expression of ITGB4 was analyzed in the lysate derived from each tumor. The same tumors were also processed to be injected into recipient nude mice at limiting dilutions (see **G**). **G** Tumor incidence at week 4 following secondary transplants of tumor-dissociated cells from mice described in (**F**). The right column indicates the estimated breast tumor-initiating/stem cell (TIC) frequencies calculated using ELDA. The overall chi-squared test for differences in TIC frequencies between groups is *p* < 0.05. **H** Images of tumors resected from nude mice (*N* = 6) that were injected at limiting dilutions as described in (**F**, **G**)

Animal experiments complied with Johns Hopkins ACUC protocols. In vitro pre-treatment (Fig. 7E): MDA-MB-231 cells were grown in fresh media containing 1 µM MBZ or DMSO (vehicle control) for 72 h. Cells were trypsinized, counted, and resuspended at 10^6^, 10^5^, 10^4^, or 10^3^ cells per 100 µL in a 1:1 ratio of Matrigel to PBS. Nude mice (female, 6–8 weeks of age) were implanted in the 2nd, 3rd, 4th, and 5th mammary fat pad on either side of the mice. The right mammary fat pads were injected with cells treated with vehicle control, and the left mammary fat pads were injected with MBZ-treated cells as follows: 2nd—10^6^ cells, 3rd—10^5^ cells, 4th—10^4^, and 5th—10^3^. In vivo pre-treatment (Fig. 7G): MDA-MB-231 cells were injected into the right and left mammary fat pads of three NSG mice. Five days post-injection, mice were treated with 30 mg/kg MBZ in a sesame oil suspension (*N* = 2) or sesame oil alone (*N* = 1) 4 × per week. After 30 days, tumors and lungs were excised for further processing. Primary tumors underwent enzymatic digestion in 2 mg/mL collagenase for 1 h at 37 °C at 160 g and strained through a 70 µm filter. Cells were counted and resuspended at 0.8 × 10^6^, 0.8 × 10^5^, 0.8 × 10^4^, or 0.8 × 10^3^ cells per 100 µL in a 1:1 ratio of Matrigel to PBS. Secondary recipient nude mice (female, 6–8 weeks of age) were implanted in the 2nd, 3rd, 4th, and 5th mammary fat pad on either side of the mice. The right mammary fat pads were injected with tumor-derived cells from NSG mice treated with sesame oil alone. In contrast, the left mammary pads were injected with the following dilutions of tumor-derived cells from NSG mice treated with 30 mg/kg MBZ in a sesame oil suspension as follows: 2nd—0.8 × 10^6^ cells, 3rd—0.8 × 10^5^ cells, 4th—0.8 × 10^4^, and 5th—0.8 × 10^3^. In both the in vitro and in vivo pre-treated LDA experiments, mouse weights and tumor growth were regularly measured. At the end of the experiment, tumors were excised and weighed. Stem cell frequency was calculated based on tumor incidence in all treatment groups using ELDA: Extreme Limiting Dilution Analysis. [35]

### *Mebendazole *in vivo* dosages*

MBZ pure polymorph C powder (Batch No. 1180916-WC, K A Malle Pharmaceuticals LTD) was utilized in all in vitro experiments and utilized for oral gavage dosing of mice. The mice were weighed on a weekly basis with their body masses averaged in treatment groups to calculate the 30 mg/kg MBZ dosage in a sesame oil (Sigma-Aldrich) suspension. The mice were treated 4× a week for the experiment and exposed to a weekly dosage of 120 mg/kg of MBZ. A second route of MBZ treatment utilized was MBZ incorporated into mouse feed. Research Diets, Inc. (New Brunswick, NJ) formulated rodent diets with 45% Kcal fat and modified version with MBZ at 215 ppm (0.18% MBZ of total mixture) and 250 ppm (0.22% MBZ of total mixture). The ingredients are as follows: protein, carbohydrate, fat, casein, l-cystine, corn starch, maltodextrin 10, sucrose, cellulose, soybean oil, lard, mineral mix S10026, dicalcium phosphate, calcium carbonate, potassium citrate, vitamin mix V10001, choline bitartrate, FD&C yellow dye #5, and red dye #40. The mice were weighed, and food consumed was monitored to calculate the dosages administered of MBZ. The mice were able to eat the feed incorporated with MBZ seven days a week and on average consumed the following dosages: 215 ppm–152 mg/kg MBZ weekly intake (25 mg/kg/day) and 250 ppm–197 mg/kg MBZ weekly intake (32 mg/kg/day). These routes of MBZ dosages differ in dose rates since the mice exposed to the feed have consistent exposure unlike the oral gavage dosed mice that are given treatments 4 × per week.

### Statistical analysis

All data are presented as mean ± standard error of the mean (SEM), and statistical analysis was performed using GraphPad Prism 9 with statistical tests appropriate for each experimental setup. Each comparison of two variables was performed using an unmatched two-way ANOVA with Bonferroni multi-comparison tests. A comparison of a single variable measured in a sample at two different locations was performed via paired two-tailed Student’s t test. A comparison of “yes” or “no” metastases categorical data was performed via a chi-squared test. Significance levels are reported as ****P < 0.0001, ***P < 0.001, **P < 0.01 *P < 0.5.

## Results

### Treatment of triple-negative breast cancer (TNBC) cells with mebendazole (MBZ) inhibited proliferation and colony formation

The ability of MBZ to decrease cell survival has been tested in thyroid, glioblastoma, breast, and colon cancer cell lines [15, 17, 23] with IC_50_ values ranging from 0.1 μM – 0.8 μM. We determined the IC_50_ values for commonly cultured BC cell lines. In mouse BC cells, MMTV-PyMT, and 4T1, the IC_50_ values were 0.39 μM and 0.58 μM, respectively. For human TNBC cell lines, MDA-MB-231, and SUM159, the MBZ IC_50_ values were 0.21 μM and 0.33 μM, respectively (Fig. 1A). This is consistent with the IC_50_ value for SUM159 cells previously reported [17] and demonstrates that the IC_50_ for BC cells is consistent with other cancer types (0.1–0.8 μM) (Additional file 2: Fig. S1A).

Next, we assessed the ability of BC cell lines treated with MBZ to form colonies using two different approaches. First, TNBC cells were pre-treated with a range of MBZ concentrations (0.01 μM, 0.05 μM, 0.1 μM, 0.5 μM, or 1 μM) or with DMSO as the vehicle control for 48 h. The cells were then seeded at 250 cells/well, and colonies were allowed to recover and form for 10–14 days (Fig. 1B, C). Then, in a second approach, BC cell lines were seeded at 250 cells/well and continually treated with a range of MBZ concentrations (0.01 μM, 0.05 μM, 0.1 μM, 0.5 μM, or 1 μM) or with a DMSO vehicle control for 10–14 days (Additional file 2: Fig. S1B-E). Whereas the first approach measured the ability for cancer cells that have survived treatment to recover and form colonies, the second approach measured the ability of cells to survive during treatment. Both methods showed that MBZ inhibited the ability of TNBC cells to form colonies. In addition, treating cells with MBZ for 48 h after initial seeding decreased cell proliferation in short-term assays (**Fig. ****1****D**; Additional file 2: Fig. S1F). Taken altogether, our results showed MBZ treatment inhibits short-term and long-term proliferation of BC cell lines and survival after treatment.

### MBZ causes the induction of G2/M cell cycle arrest in human TNBC cells and apoptosis

MBZ has previously been reported to induce G2/M cell cycle arrest and apoptosis in thyroid, glioblastoma, breast, and colon cancer cell lines [16, 17, 26, 27, 29]. Therefore, we investigated the effect of MBZ treatment on proteins involved in cell cycle arrest and apoptosis. In MDA-MB-231 and SUM159 cell lines, MBZ induced the cyclin-dependent kinase inhibitors p21 and p27. In addition, MBZ treatment increased G2/mitotic-specific cyclin-B1 (CCNB1) and phosphorylated cyclin-dependent kinase 1 (CDK1), indicative of a G2/M cell cycle arrest (Fig. 2A). Flow cytometry analysis of propidium iodide-stained MDA-MB-231 cells confirmed that cells undergo a dose-dependent G2/M cell cycle arrest following MBZ treatment (Fig. 2B; Additional file 2: Fig. S2A, B). We also observed that MBZ treatment increased the expression of the apoptotic marker, cleaved PARP (poly ADP-ribose polymerase), and DNA damage marker, phosphorylated gamma-H2A histone family member X (γH2AX) (Fig. 2C). Likewise, there was a sixfold increase in dead cells following treatment with DMSO or 1 μM MBZ (Additional file 2: Fig. S2C). Altogether, the results demonstrated that, like other cancer types, MBZ induces G2/M cell cycle arrest in TNBC cells following 48 h of treatment and apoptosis after 72 h of treatment.

### In vitro* MBZ reduces human TNBC cell migration*

In addition, MBZ has been shown to reduce the migration and invasion of thyroid and gastric cancer cells [16, 28] and more recently in MDA-MB-231 cells [29]. Therefore, we used two independent assays to determine whether MBZ could also inhibit the migration of TNBC cells. First, we performed a wound-healing assay to assess the ability of MBZ to inhibit cell migration in vitro. SUM159 and MDA-MB-231 cells were grown to confluence, and a single scratch (or wound) was generated (0 h). The cells were treated with MBZ at a concentration of 0.25 μM, 0.35 μM, 0.5 μM, or with DMSO as vehicle control. The wounds were imaged at time 0 h, 12 h, and 24 h to calculate the average area of the wound covered with cancer cells. Compared to the vehicle control, MBZ treatment significantly decreased the average healing speed of the wound starting 12 h post-treatment with a 0.35 μM MBZ concentration in MDA-MB-231 cells (Fig. 3A, B). In a second migration assay, MBZ reduced the ability of SUM159 and MDA-MB-231 cells to migrate through Transwell pores following 48 h of pre-treatment with 0.5 μM and 1 μM of MBZ as compared to DMSO (Fig. 3C, D). Given the correlation between migratory ability and metastasis [36], our results suggest that even if a cell can survive MBZ treatment, it has a reduced capacity to migrate, and likely a decreased ability to metastasize.

### MBZ reduces primary tumor growth and prevents distant organ metastases in an orthotopic model of TNBC

Previous studies have shown that orally administered MBZ reduced metastasis in orthotopic models of thyroid cancer [16] and adrenocortical carcinoma [37], respectively. Given these findings and the reduction in cell migration, we reasoned that MBZ would also reduce the metastatic spread of TNBC cells. We established orthotopic tumors by injecting fluorescently tagged MDA-MB-231 cells in the mammary fat pad of female NSG (NOD-SCID Gamma) mice. Five days post-injection, the mice were randomized and placed in the following treatment groups: sesame oil as a vehicle gavage and a high-fat KetoCal diet (Con; *N* = 12), 215 ppm MBZ incorporated into a high-fat KetoCal diet (Feed; *N* = 7), 20 mg/kg MBZ in a sesame oil gavage (20 mg/kg; *N* = 6; 4 times per week), or 30 mg/kg MBZ in sesame oil gavage (30 mg/kg; *N* = 12; 4 times per week). Mice treated with either 215 ppm MBZ feed or 30 mg/kg MBZ sesame oil gavage had slower tumor growth and 35% or 55% smaller primary tumors at the endpoint of the study as compared to control mice, respectively (Fig. 4A, B; Additional file 2: Fig. S3A, B). MBZ decreased metastatic burden by 60% and 72% as measured by quantifying the amount of human DNA content in mouse lungs for both the 215 ppm MBZ fed group and the group treated with 30 mg/kg MBZ by oral gavage as compared to the control group, respectively (Fig. 4C). The results suggested that MBZ has an even more dramatic effect on metastasis than reduction in tumor growth.

To more closely recapitulate clinical protocols used to treat patients with BC, mice received 215 ppm MBZ incorporated into their feed using a neoadjuvant treatment protocol. Five days after cell implantation, mice were placed on MBZ feed. Tumors were surgically resected 5 weeks post-implantation and adjuvant MBZ treatment continued for two additional weeks. No toxicities were associated with the MBZ feed or surgery throughout the experiment as assessed by mouse body weight (Additional file 2: Fig. S3C). Still, the MBZ-fed mice had slower tumor growth and significantly smaller primary tumors at the time of resection (Fig. 4D, E). At the endpoint of the experiment, we extracted genomic DNA from mouse lungs and livers to measure metastatic spread using qPCR to quantify human HK2 DNA content. Mice treated with 215 ppm MBZ feed had significantly reduced lung (Fig. 4F; Additional file 2: Fig. S3D) and no detectable liver metastases (Fig. 4G; Additional file 2: Fig. S3E). To rule out the effect of primary tumor size contributing to the difference in metastasis, we normalized HK2 DNA content by individual tumor weight and compared it to the mean of the control group. The results demonstrated that MBZ reduces lung metastasis to a greater extent than it inhibits tumor growth and MBZ completely abolishes liver metastases.

We expanded our preclinical models by orthotopically implanting a TNBC patient-derived xenograft (PDX), HCI-001, in NSG mice. Following 8 weeks of growth, the primary tumors reached an average tumor volume of ~ 150 mm^3^ (Additional file 2: Fig. S3F, G). The mice were then randomized into two treatment groups which were dosed 4 times per week: sesame oil as a vehicle gavage (Con, *N* = 8) or 30 mg/kg MBZ in sesame oil gavage (30 mg/kg; *N* = 9). After two weeks of treatment, there was no decrease in the size of tumors for the MBZ group nor a reduction (Fig. 4H; Additional file 2: Fig. S3H, I) of tumor weight (Fig. 4I). However, we did observe a twofold decrease in metastatic burden in the lung (Fig. 4J). We further confirmed a significant decrease in lung metastases by counting the number of nodules present in H&E stained mouse lungs. We observed a twofold reduction in lung nodules in mice treated with 30 mg/kg MBZ compared to mice given vehicle control (Fig. 4K, L; Additional file 2: Fig. S3J). The results highlight the ability of MBZ to reduce lung metastases in a TNBC PDX model, which has clinically relevant implications since PDX models closely match patients’ therapeutic responses. [38]

### MBZ decreases primary tumor growth and diminishes distant organ metastases in a syngeneic model of TNBC

Next, we tested the ability of MBZ to inhibit growth and metastasis of the 4T1 BC cell line that was derived from the mammary gland tumor of immune-competent, BALB/c mouse. 4T1-luciferase-tagged cells were orthotopically implanted into the mammary fat pad of mice. MBZ decreased primary tumor growth. The final tumor weight was decreased by 2.6-fold in MBZ-fed mice compared to the control mice without toxicity (Fig. 5A, B; Additional file 2: Fig. S4A, B). In addition, in vivo bioluminescence images taken at several time points throughout the experiment illustrated that the tumors in MBZ-fed mice had decreased bioluminescence, indicating that they grew at a slower rate (Fig. 5C, D; Additional file 2: Fig. S4C). At the endpoint of the experiment, MBZ reduced lung bioluminescence by 6.5-fold compared to the control mice (Fig. 5E, F). To confirm our bioluminescence results, we also measured the luciferase DNA content in the lungs (Fig. 5G). Strikingly, bioluminescence could not be detected in the liver of MBZ-fed mice, confirming our previous observations (Fig. 5H, I). Overall in three in vivo mouse models of TNBC, MBZ reduced primary tumor growth by 2–threefold, lung metastasis by 2–sixfold (in four in vivo models), and completely eliminated liver metastasis.

#### *MBZ reduces the cancer stem cell regulator, Integrin*β*4, in TNBC cell lines*

To uncover the main gene expression changes altered by MBZ treatment, we performed RNA sequencing of MDA-MB-231 and SUM159 cells following 72 h treatment of 1 μM MBZ compared to DMSO vehicle control. A total of 420 genes were upregulated (FC ≥ 1.5), and 140 genes were downregulated (FC ≤ 1.5) by MBZ treatment compared with cells treated with DMSO in both cell lines (Fig. 6A). Using a gene ontology (GO) enrichment analysis, we confirmed MBZ downregulated pathways that were involved in angiogenesis in TNBC cells as has been previously reported for medulloblastoma, colon cancer, and thyroid cancer (Additional file 2: Fig. S5A) [16, 23, 39]. A principal component analysis showed that the trajectory of gene expression changes along PC3 was the same in for both MDA-MB-231 and SUM159 cells following treatment with MBZ (Additional file 2: Fig. S5B).

Due to the dramatic reduction in distant organ metastases in our in vivo TNBC mouse models, we focused on the most differential expression genes that were down regulated with MBZ treatment and that have also been previously reported to prevent or decrease BC metastases in animal models (Fig. 6B). We uncovered four well-known drivers of metastases whose expression was reduced following MBZ treatment: members of the transforming growth factor β (TGFβ) family, SRY-related HMG box 4 (SOX4), lysyl oxidase like 1 (LOXL1), and Integrin β4 (ITGβ4).

We used RT-qPCR to confirm the ability of MBZ treatment to reduce the expression of the candidate genes (Fig. 6B). Only ITGβ4 gene expression was decreased in both cell lines following 72 h of MBZ treatment (Fig. 6C, D). To confirm this result at the protein level, ITGβ4 was measured in cells by flow cytometry analysis and Western blot analysis (Fig. 6E, F; Additional file 2: Fig. S5C, D). This reduction occurred dose-dependently beginning at concentrations as low as 0.05 μM in MDA-MB-231 cells and 0.125 μM in SUM159 cells (Additional file 2: Fig. 5C). In addition, MDA-MB-231 cells that survived 0.5 μM of MBZ (Sytox negative) had a 19-fold increase in the ITGβ4-negative population (Additional file 2: Fig. S5E).

Previously, ITGβ4 has been shown to be a marker of cancer stemness [40]. To determine whether MBZ treatment reduced the ITGβ4-positive TNBC population in MDA-MB-231 and SUM159 cells and reduced the population of cells expressing the conventional cancer stem cell markers (CSC) (CD44^hi^CD24^low^), we analyzed the percent of CD44^hi^CD24^low^ cells within the ITGβ4-positive population. We determined that MBZ reduced the cancer stem cell population by almost twofold in MDA-MB-231 cells and fourfold in SUM149 cells following 72 h of 1 μM MBZ treatment (Fig. 6G, H).

Next, we aimed to determine whether ITGβ4 expression was affected in tumors from MBZ-treated mice. Using RNA from the PDX tumors from mice treated as described in Fig. 4H, we determined that MBZ treatment led to a twofold decrease in ITGβ4 RNA expression compared to vehicle control-treated mice (Additional file 2: Fig. S6F). Next, we immunofluorescently labeled ITGβ4 protein in primary tumors of mice bearing MDA-MB-231 tumors and treated them as described in Fig. 4A, D. ITGβ4 was reduced by threefold (Fig. 6I; model first described in Fig. 4A) or twofold (Fig. 6J; model first described in Fig. 4D) in NSG mice treated with MBZ compared to vehicle control (Additional file 2: Fig. S5G). Overall, MBZ treatment in vitro and in vivo consistently reduced ITGβ4 RNA and protein expression.

### MBZ treatment reduces the cancer stem cell phenotype of TNBC cells

Previously, Zhang et al*. *[17] determined MBZ treatment in vitro reduced the number of mammospheres formed by SUM159 and MDA-MB-231 cells treated with MBZ versus DMSO. Similarly, we found that MBZ reduced mammosphere formation of MDA-MB-231 cells by 40% when treated with 0.25 μM MBZ compared to DMSO (Fig. 7A, B; Additional file 2: Fig.S6A). We also demonstrated that ITGβ4+ tumor-derived MDA-MB-231 cells formed 4× more mammospheres than ITGβ4-cells (Additional file 2: Fig. S6B), supporting its role as a stem cell marker as previously shown [40]. We observed a 15% reduction in the ability for tumor-derived MDA-MB-231 cells from NSG mice treated for 30 days with MBZ to form mammospheres compared to tumor-derived cells from a vehicle control-treated mouse (Fig. 7C; Additional file 2: Fig. S6C).

Next, we utilized a limiting dilution assay to evaluate the ability of MDA-MB-231 cells treated with mebendazole in vitro or in vivo to form tumors in nude mice (Fig. 7D). MDA-MB-231 cells were pre-treated in vitro for 72 h with DMSO alone or treated with 1 μM MBZ. After treatment, the viable cells were injected at limiting dilutions into nude mice. DMSO-treated cells had a tumor-initiating cell (TIC) frequency of 1/17,169 cells, whereas cells treated with MBZ did not develop tumors at any dilution (Fig. 7E; Additional file 2: Fig. S6D–G). The drastic reduction in self-renewal capabilities in cells pre-treated with MBZ in vitro prompted a second limiting dilution assay (LDA). NSG mice bearing MDA-MB-231 cell-derived tumors were treated for 30 days with 30 mg/kg MBZ or sesame oil alone four times per week. As previously shown, MBZ treatment reduced tumor growth, ITGβ4 expression, and lung metastasis (Fig. 7F; Additional file 2: Fig. S6H–J). Likewise, the tumor-derived MDA-MB-231 cells treated for 30 days with a vehicle control were more efficient at tumor initiation, with a TIC frequency of 1/45,059 compared to the TIC frequency of 1/188,578 from tumor-derived MDA-MB-231 cells treated with MBZ (Fig. 7G). The fourfold difference in TIC frequency corresponded with decreased tumor burden at each injection site (Fig. 7H; Additional file 2: Fig. S6K–M). The data suggested that cells that survive MBZ treatment in the primary tumor have reduced ITGβ4 expression and a decreased ability to form mammospheres and initiate tumor formation.

## Discussion

Our preclinical studies demonstrated that the anthelminthic, MBZ, decreased the growth of TNBC and significantly abrogated lung metastasis while eliminating liver metastases. We showed that MBZ decreased TNBC cell proliferation through previously described mechanisms of action, including G2/M cell cycle arrest and apoptosis [16]. Using RNA sequencing, we discovered that MBZ also reduces ITGβ4 expression and previous studies have linked ITGβ4 expression with increased metastasis [41]. Likewise, targeting ITGβ4 with ITGβ4 protein-pulsed dendritic cell vaccination or via an anti-CD3/anti-ITGβ4 bispecific antibody decreased metastasis in mouse models of BC [42]. ITGβ4 can be used to stratify TNBC cells that differ in their tumor initiation abilities and has been used as a cancer stem cell marker to identify more aggressive subtypes of TNBC [40]. Our studies show that MBZ decreases ITGβ4 expression concomitant with decreases in cell stemness in vitro and in vivo as measured by mammosphere formation and limiting dilution assays. Taken together, the results suggest that a reduction in ITGβ4 expression may contribute to the effectiveness of MBZ in reducing metastasis.

A recent high throughput screening study identified MBZ as a therapeutic that could inhibit radiation-induced de-differentiation in the TNBC cell line, SUM159. In this study, MBZ had impressive in vitro efficacy but only a modest in vivo efficacy (10 mg/kg or 20 mg/kg I.P. for 3 weeks) when used as a single agent [17]. In our study, we showed that MBZ decreased TNBC cell proliferation and viability using human (SUM159, MDA-MB-231) and mouse (4T1, MMTV-PyMT) BC cell lines. We show that MBZ has significantly greater therapeutic efficacy than in the Zhang et al. study. This may be due in part to differences in the method of delivery of MBZ. We delivered MBZ polymorph C orally by incorporating it into a high-fat diet or using a high-fat suspension such as sesame oil. Previous studies have shown that this delivery method increased the absorption of MBZ. Additionally, Polymorph C has higher absorption than other polymorphs that have been tested in mouse models [16, 19, 20].

TNBC is the most challenging BC subtype to treat and results in higher mortality due to the high incidence of metastatic cases and paucity of treatment options. Most patients with BC would survive their disease if metastasis could be prevented. Our preclinical results showed that MBZ reduced metastasis with no side effects in mice, and it is well tolerated in humans even at higher doses and for longer durations than used in mice [27]. Given this safety profile, MBZ is a candidate for long-term use and may have promise as adjuvant therapy following surgical resection to prevent BC metastasis. Future studies considering the addition of MBZ to the standard of care treatment are warranted in order to advance MBZ in clinical trials for patients with breast cancer.

## Supplementary Information

**Additional file 1**. List of antibodies and primers used in the study.

**Additional file 2**. Supplementary Figures.

**Additional file 3**. Differential Expression Data.

## Data Availability

The data generated in this study are available at in Gene Expression Omnibus (GEO) at GSE190845.
