# Supplementary material for: Mebendazole prevents distant organ metastases in part by decreasing ITGβ4 expression and cancer stemness
Source: Breast Cancer Res. 2022 Dec 28;24:98. doi: 10.1186/s13058-022-01591-3 (PMC9798635; doi:10.1186/s13058-022-01591-3)
Supplement: Supplementary file 1 — Additional file 1. List of antibodies and primers used in the study. [file 13058_2022_1591_MOESM1_ESM.docx]

**Supplementary Table 1. Antibodies used for immunoblot experiments**

**Antibodies**

| Protein | Dilution | Company | Catalog# | WB/IF/Flow |
| --- | --- | --- | --- | --- |
| CCNB1 | 1:1000 | Santa Cruz Biotechnology | SC-245 | WB |
| CCND1 | 1:1000 | Cell Signaling Technology | 2922S | WB |
| CCNE1 | 1:1000 | Cell Signaling Technology | 4129S | WB |
| p-CDK1 | 1:1000 | Abclonal | AP0016 | WB |
| CDK1 | 1:1000 | Abclonal | A0220 | WB |
| p21 | 1:1000 | Cell Signaling Technology | 2947S | WB |
| p27 | 1:1000 | Cell Signaling Technology | 3686S | WB |
| 𝛃-actin | 1:10,000 | Proteintech | #HRP-60009 | WB |
| Uncleaved and Cleaved PARP | 1:1000 | Abclonal | A19596 | WB |
| p-ƔH2A.x | 1:1000 | Cell Signaling Technology | 9718S | WB |
| Histone H2AX | 1:1000 | Abclonal | A11361 | WB |
| Integrin𝛃4 | 1:1000 | Abclonal | 1032129 | WB |
| Integrin𝛃4 (CD104) | 1:100  1:300 | ThermoFisher | 50-1049-80 | IF  Flow |
| CD44 | 1:300 | Biolegend | 397517 | Flow |
| CD24 | 1:300 | Biolegend | 983602 | Flow |

**Supplementary Table 2. Primers used for RT-PCR experiments**

**Primers**

| Gene | Forward Primer (5’-3’) | Reverse Primer (5’-3’) | |
| --- | --- | --- | --- |
| ITG𝛃4 (human) | GCTTCACACCTATTTCCCTGTC | | GACCCAGTCCTCGTCTTCTG |
| Firefly Luciferase | GTGGTGTGCAGCGAGAATAG | | CGCTCGTTGTAGATGTCGTTAG |
| Hexokinase 2 (human) | CCAGTTCATTCACATCATCAG | | CTTACACGAGGTCACATAGC |
| 18S (human) | CCAGTTCATTCACATCATCAG | | CTTACACGAGGTCACATAGC |
