# Supplementary material for: Mebendazole prevents distant organ metastases in part by decreasing ITGβ4 expression and cancer stemness
Source: Breast Cancer Res. 2022 Dec 28;24:98. doi: 10.1186/s13058-022-01591-3 (PMC9798635; doi:10.1186/s13058-022-01591-3)
Supplement: Supplementary file 2 — Additional file 2. Supplementary Figures. [file 13058_2022_1591_MOESM2_ESM.pdf]

Supplementary Figure 1

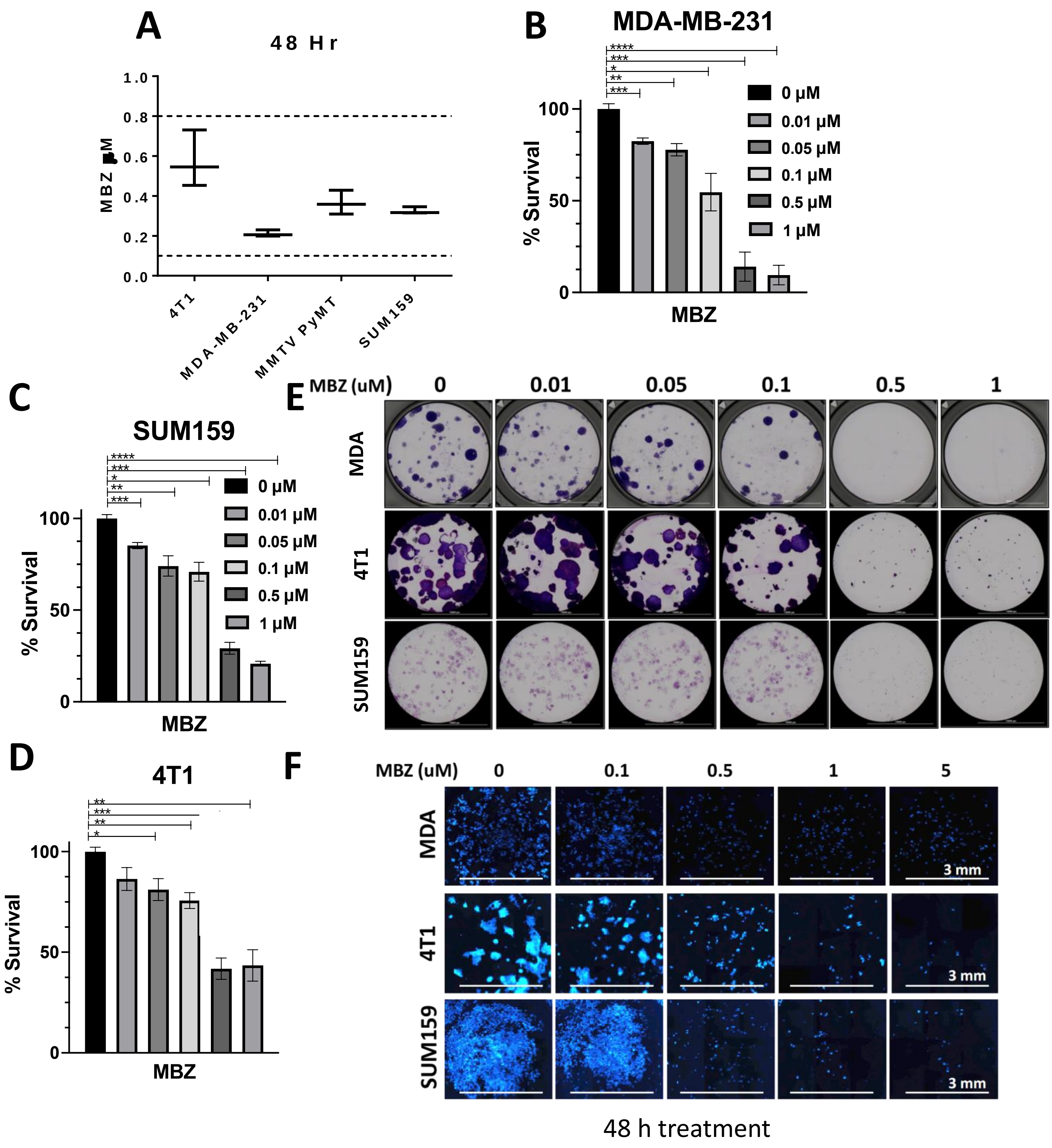

Supplementary Figure 1. Mebendazole inhibits the proliferation of triple-negative breast cancer cells. (**A)** The half-maximal inhibitory concentration (IC_{50}) of MBZ in MMTV PYMT, MDA-MB-231, 4T1, and SUM159 cells is plotted with SEM for 3 independent experiments. Dotted lines display the range of IC_{50} values (0.1-0.8 µM) as published in the literature for other cancer cell lines (see: Guerini *et al*. 2019 and Pantziarka *et al.* 2014). **(B-D)** Quantification of colony formation assays from MDA-MB-231 (B), SUM159 (C), or 4T1 cells shown in Supplementary Fig. 1E. Mean ± SEM; N = 3 independent experiments with n=4 technical replicates. P-values for one-way ANOVA test *<0.05,**<0.01,***<0.001,****<0.0001.(**E**) Representative images of colonies formed from MDA-MB-231 (top), 4T1 (middle), and SUM159 (bottom) cells that were seeded at 250 cells per well, treated at varying concentrations (0 µM, 0.01 µM, 0.05 µM, 0.1 µM, 0.5 µM, and 1 µM), incubated for 10-14 days in growth media and stained with crystal violet. (**F**) A representative image of DAPI staining in SUM159 (bottom), 4T1 (middle), and MDA-MB-231 (top) cells.

Supplementary Figure 2

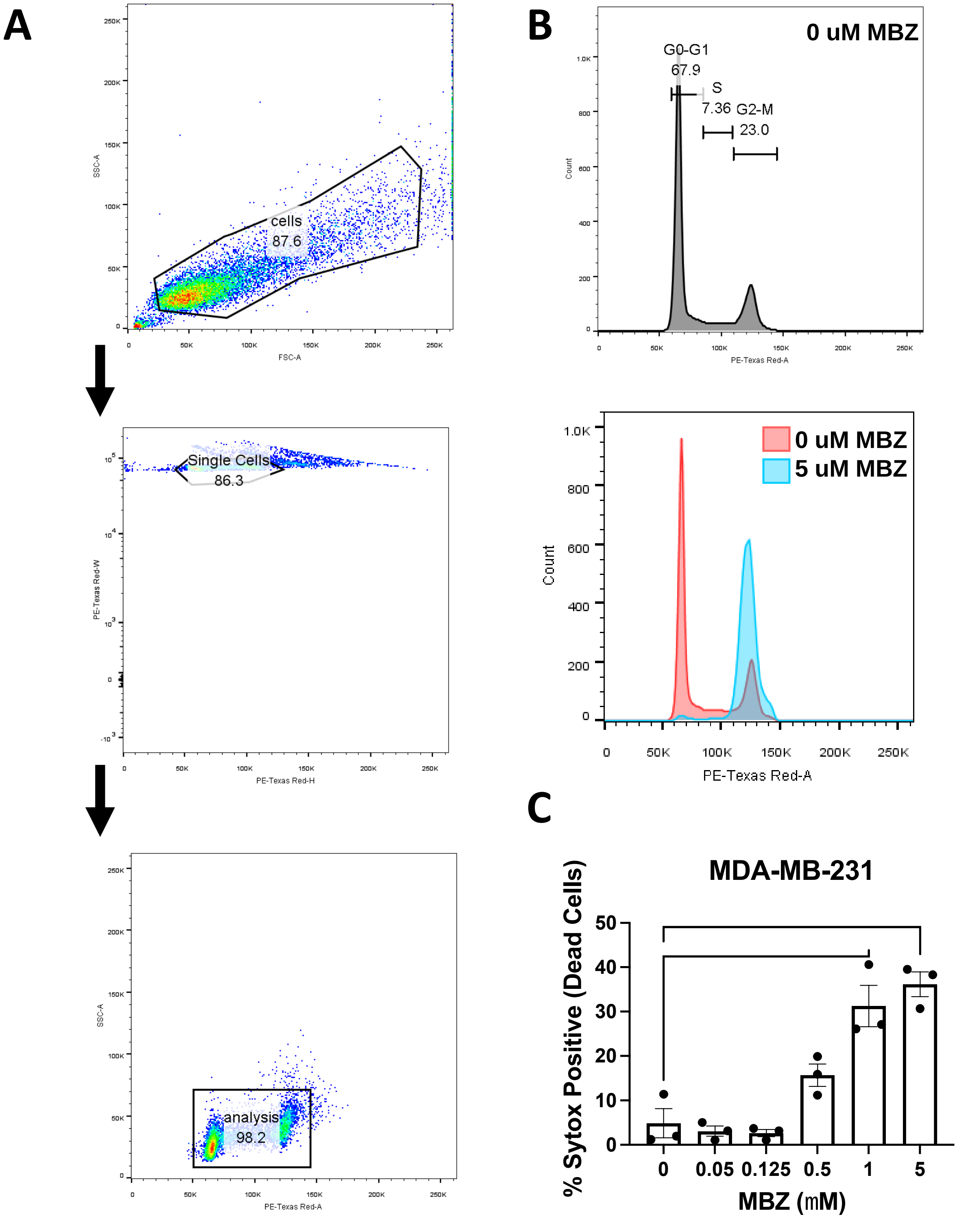

Supplementary Figure 2. Mebendazole induces G2/M cell cycle arrest and apoptosis. (**A**) The gating strategy for flow cytometry experiments conducted with MDA-MB-231 cell lines in Figure 2B. (**B**) Cell cycle histograms for MDA-MB-231 cells stained with propidium iodide after 48h of treatment with 5 μM MBZ or with DMSO as a vehicle control. (**C**) Quantification of the % of Sytox positive cells as a measurement of dead cells for MDA-MB-231 cells (see also Supplemental Figure 5D) treated with 0.05 μM, 0.125 μM, 0.5 μM, 1 μM and 5 μM MBZ or with DMSO as a vehicle control for 72h.

Supplementary Figure 3

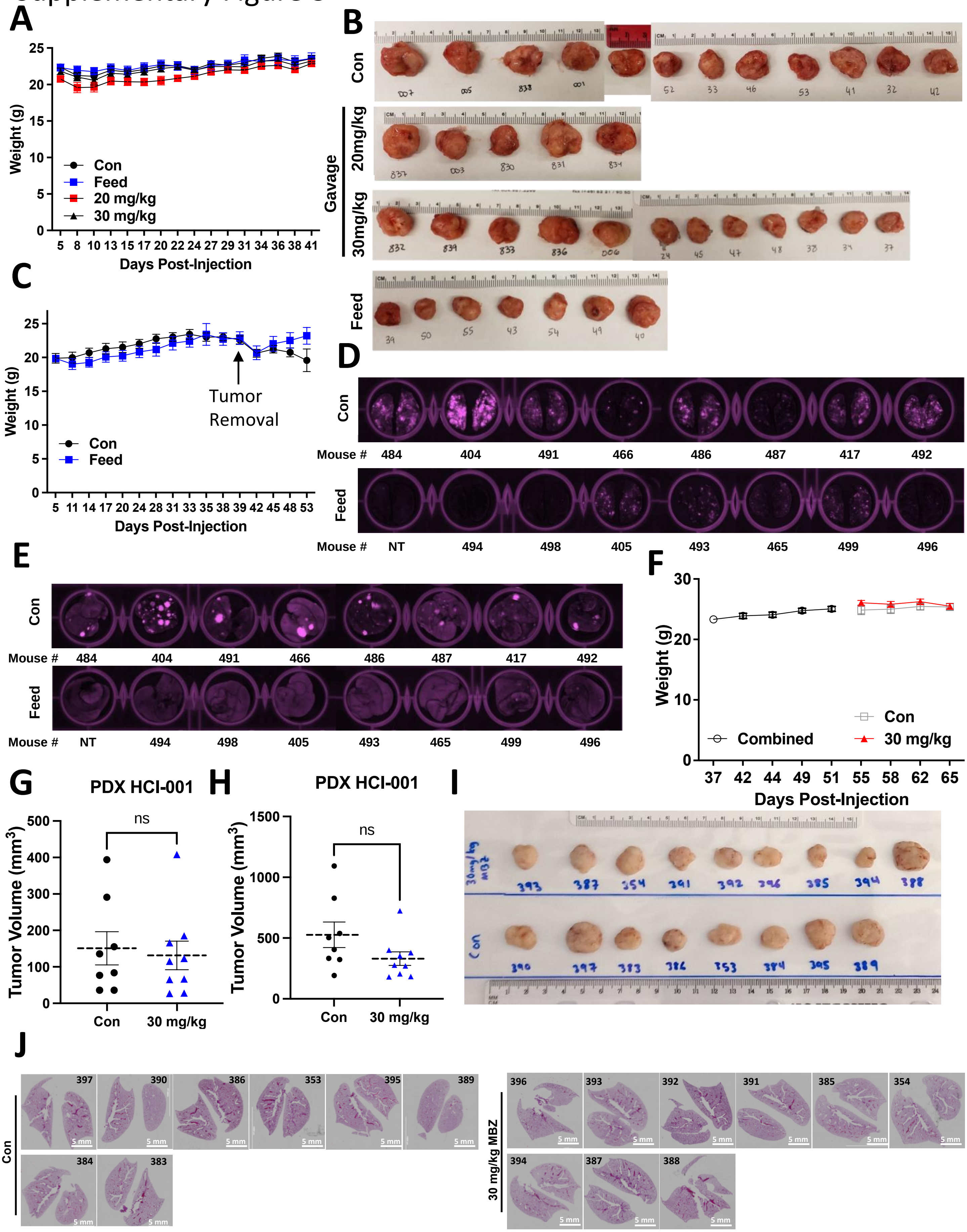

Supplementary Figure 3. Mebendazole reduces lung metastases. (**A**) Mouse weight measured over time and across treatment groups as indicated in Figure 4A (mean ± SEM, N = 6-12 mice). (**B**) Images of tumors resected from NSG mice that were treated as described in Figure 4A. (**C**) Mouse weight measured over time and across treatment groups as indicated in Figure 4D (mean ± SEM, N = 9-12 mice). (**D-E**) Representative fluorescent images of lungs (D) or livers (E) resected at the endpoint of the experiment as described in Figure 4C prior to DNA extraction. (**F**) Mouse weight measured over time and across treatment groups as indicated in Figure 4H-M (mean ± SEM, N = 8-9 mice). (**G**) Tumor volume of NSG primary tumors established with tumor-derived HCI-001 PDXs that were manually and enzymatically processed. Two-way *t* test. (**H**) Tumor volume of HCI-001 PDXs two weeks after mice were treated with either 30mg/kg MBZ in sesame oil (N=9) or vehicle control (N=8). Two-way *t* test. (**I**) NSG mice bearing HCI-001 PDXs were treated with sesame oil (N=8) or 30 mg/kg of MBZ in sesame oil (N=9). Images of each tumor at the time of resection. (**J**) Representative lung sections H&E stained from NSG mice bearing primary HCI-001 PDXs (described in Figure 4K).

Supplementary Figure 4

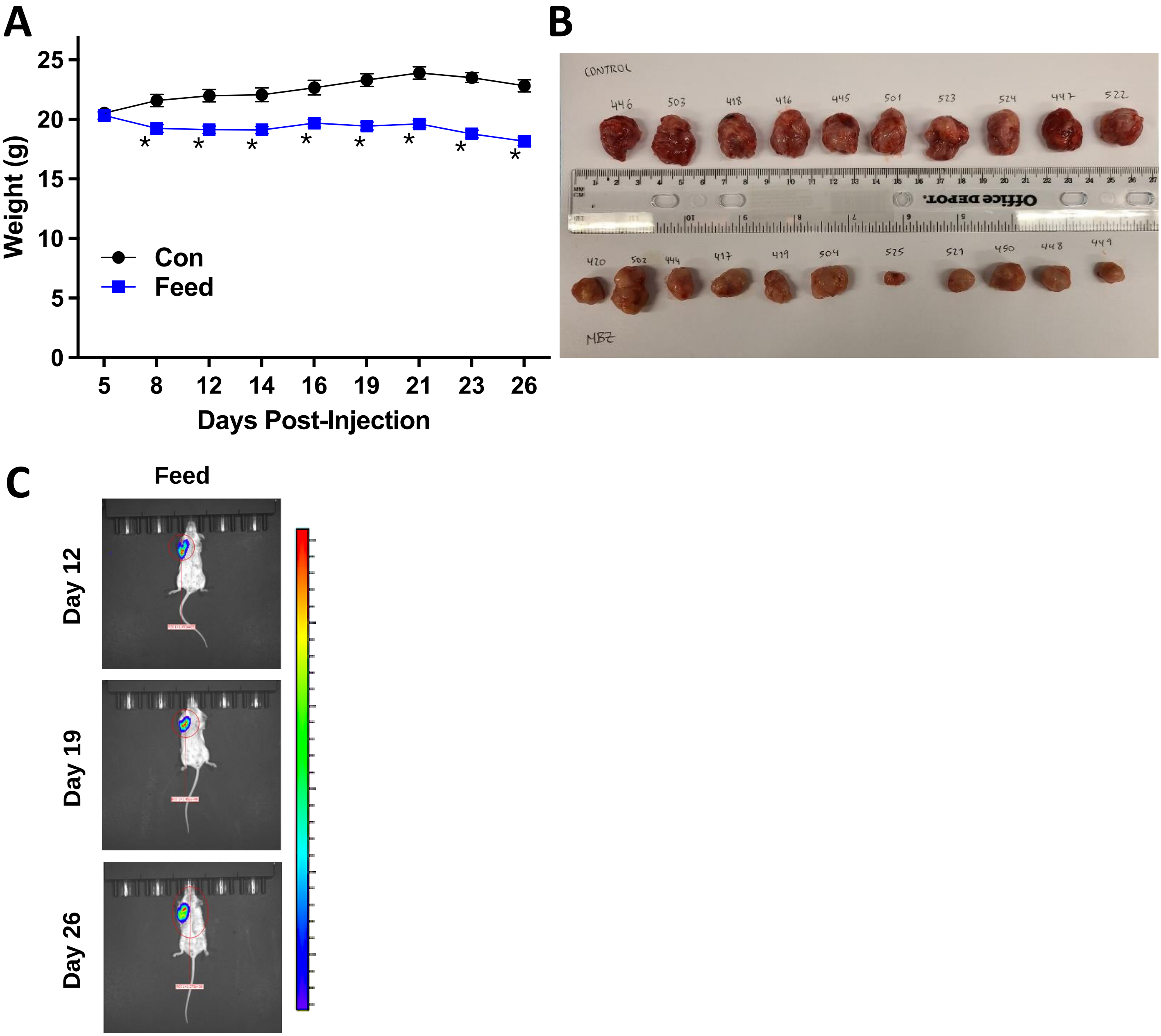

Supplementary Figure 4. Mebendazole reduces tumor growth and lung and liver metastases in an immunocompetent animal model of metastasis. (**A**) Balb/c mouse weight over time and across each treatment group (mean ± SEM, N = 10-11 mice). Treatments began five days post left mammary fat pad injection of 500 luciferase-tagged 4T1 cells ; Control –high fat KetoCal diet N=10, Feed – 250 ppm MBZ high fat KetoCal diet N=11. P-values for two tailed *t* test *<0.05. (**B**) Images of final tumors from Balb/c mice. (**C**) Full body bioluminescence imaging of a mouse in the feed group not shown in main figure.

Supplementary Figure 5

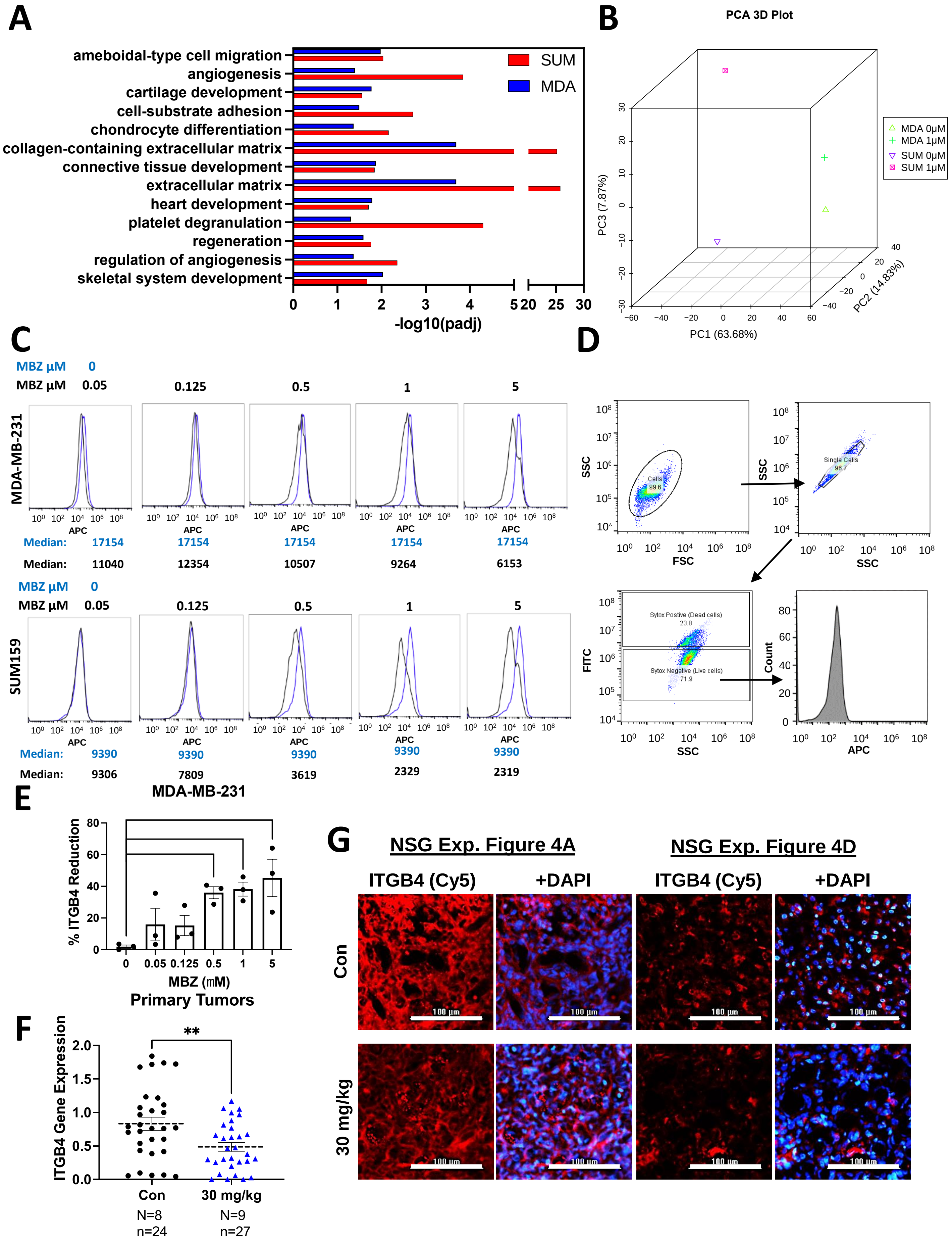

Supplementary Figure 5. Mebendazole treatment reduces ITGβ4 expression. (**A**)The -log10 adjusted p-values of GO (gene ontology) gene analysis pathways in MDA-MB-231 and SUM159 cells treated with 1 μM of MBZ or with DMSO as a vehicle control for 72h. (**B**) 3D PCA plot derived from the quantification analysis obtained from RNAseq data of MDA-MB-231 cells and SUM159 cells treated with 1 μM or with DMSO as a vehicle control for 72h. (**C**) Flow cytometry histograms for MDA-MB-231 and SUM159 cells stained with ITGβ4 antibody after 72 h of treatment with 0.05 μM, 0.125 μM, 0.5 μM, 1 μM and 5 μM MBZ or with DMSO as a vehicle control. The APC peak median values are listed below each histogram for each cell line shown in Fig. 6E. (**D**) The gating strategy used for flow cytometry experiments conducted with MDA-MB-231 and SUM159 cell lines co-stained with ITGβ4 and Sytox in Fig. 6C-D and Supplementary Fig. 6C-D. (**E**) The % reduction in ITGβ4 expression in MDA-MB-231 cells as detected by flow cytometry following 72h of treatment with 0.05 μM, 0.125 μM, 0.5 μM, 1 μM, 5 μM MBZ or with DMSO as a vehicle control. P-values for one-way ANOVA test *<0.05, **<0.01. (**F**) Quantification of ITGB4 gene expression using RT-qPCR performed with RNA extracted from NSG primary HCI-001 PDX tumors in both treatment groups (Con or 30 mg/kg MBZ) normalized to the human 18S gene. Mean ± SEM; Control (N=8 mice) and 30 mg/kg MBZ (N=9 mice) with n=3 technical replicates per primer. P-values for two-way unpaired *t* test **<0.01. (**G**) Representative fluorescent images of NSG primary tumors either treated with 30 mg/kg MBZ in sesame oil suspension via gavage or vehicle control stained with a fluorescently tagged ITGβ4 antibody (red) and DAPI (blue). The images were analyzed and quantified in Figure 6I-J.

Supplementary Figure 6

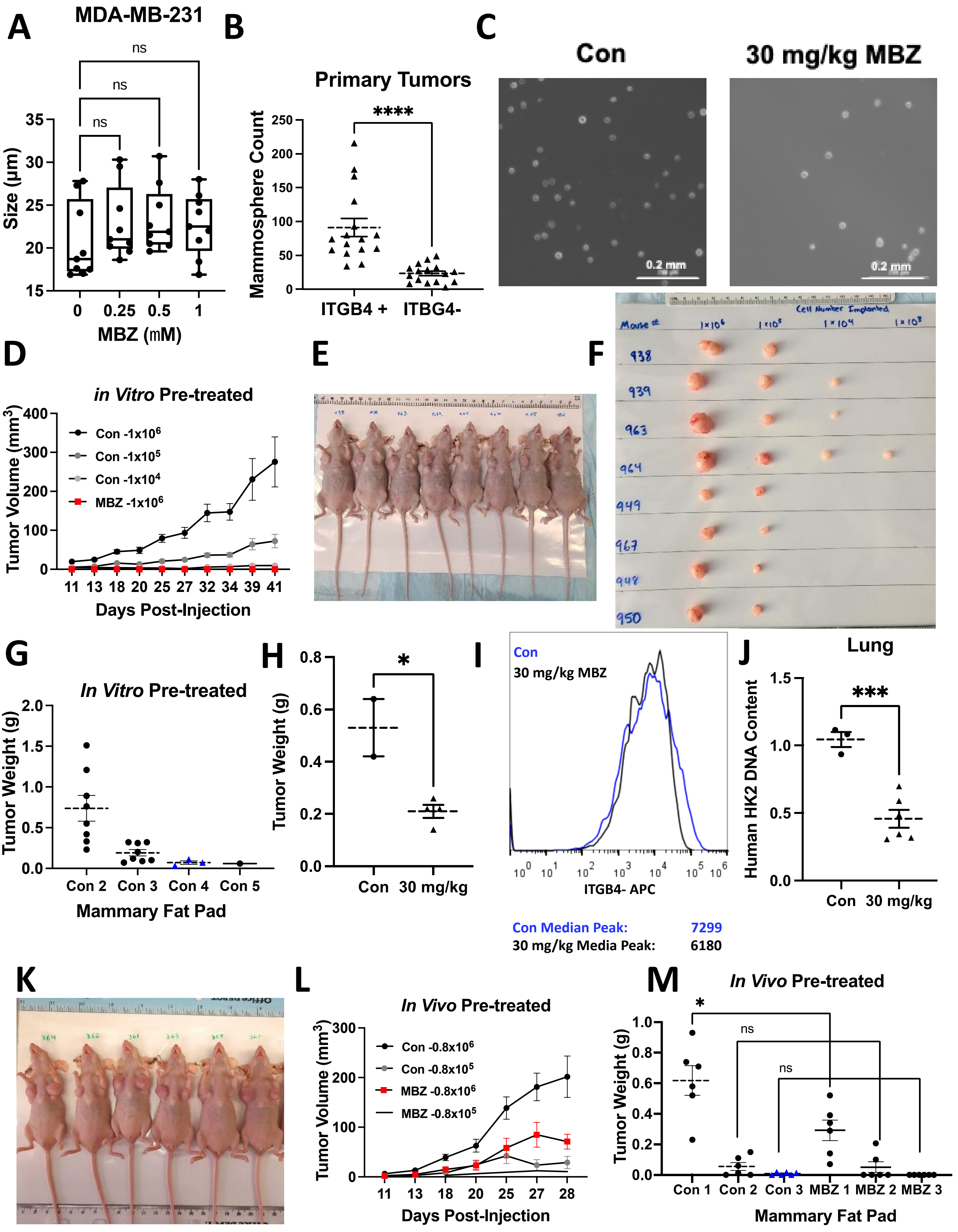

Supplementary Figure 6. Mebendazole treatment reduces the cancer stem cell properties of TNBC cells. (**A**) Quantification of the size of mammospheres that were formed by MDA-MB-231 cells after 72h pre-treatment with MBZ (0.25 μM, 0.5 μM, and 1 μM) or DMSO as a vehicle control. Mean ± SEM; N = 3 independent experiments with n = 3 technical replicates. One-way ANOVA test. (**B**) Quantification of the total number of mammospheres that were formed by tumor-derived MDA-MB-231 cells that were Sytox-negative and either ITGβ4-postive or ITGβ4-negative after 7 days in mammosphere formation media. Mean ± SEM; N = 3 experiments with n=5 technical replicates. P-values for two-way *t* test ****<0.0001. (**C**) Representative images of mammospheres quantified in Figure 7C. (**D**) Tumor volume of primary tumors established with $1x10^6$, $1x10^5$, or $1x10^4$ MDA-MB-231 cells pre-treated *in vitro* with 1 μM MBZ or DMSO. (**E**) Image of all mice bearing tumors included in the *in vitro* pre-treated limiting dilution assay. (**F**) Images of tumors resected from Nude mice (N=8) that were injected with limiting dilutions as described in Figure 7E. (**G**) Tumor weights of primary tumors established with $1x10^6$, $1x10^5$, or $1x10^4$ MDA-MB-231 cells pre-treated *in vitro* with 1 μM MBZ or DMSO at week 6. (**H**) The final weight of tumors excised from NSG mice treated with either 30mg/kg of MBZ in sesame oil or vehicle control. The tumors were also processed to be injected at limiting dilutions into Nude mice. (**I**) Flow cytometry histograms for tumor-derived MDA-MB-231 cells derived from mice treated with either 30mg/kg MBZ in sesame oil or sesame oil alone (vehicle control). The tumor-derived cells were stained with ITGB4 primary antibody and the APC peak median values are listed below the overlaid histogram. (**J**) Metastatic burden for mice treated with either 30mg/kg MBZ in sesame oil or sesame oil alone was determined by measuring human genomic HK2 DNA content in mouse lungs normalized to each individual tumor weight and then normalized to the average of the Con group. The same tumors were also utilized in the *in vivo* pre-treatment limiting dilution assay (Figure 7G-H) (mean ± SEM N = 1-2 mice with n=3 technical replicates). P-values for two tailed *t* test ***<0.001. (**K-M**) Images (K), tumor volume (L) and tumor weight (M) of secondary tumors established with $0.8x10^6$,$0.8x10^5$ or $0.8x10^4$ tumor-dissociated cells from tumors initially formed in mammary fat pads of NSG mice implanted with MDA-MB-231 cells. Mice were treated with 30mg/kg MBZ in sesame oil (MBZ) or sesame oil alone (CON) by oral gavage. (2- 2nd mammary fat pad, $0.8x10^6$ tumor-derived cells; 3- 3rd mammary fat pad, $0.8x10^5$ tumor-derived cells; 4- 4th mammary fat pad, $0.8x10^4$ tumor-derived cells). P-values for two-way paired *t* test *<0.05.
